# Estimating relative risks and risk differences in randomised controlled trials: a systematic review of current practice

**DOI:** 10.1186/s13063-024-08690-w

**Published:** 2025-01-02

**Authors:** Jacqueline Thompson, Samuel I. Watson, Lee Middleton, Karla Hemming

**Affiliations:** Department of Applied Health Sciences, College of Medicine and Health, University of Birmingham, Edgbaston, West Midlands, B15 2TT UK

**Keywords:** Binary outcomes, Relative risk, Risk difference, Covariate adjustment, Statistical efficiency, Statistical practice

## Abstract

**Background:**

Guidelines for randomised controlled trials (RCTs) recommend reporting relative and absolute measures of effect for binary outcomes while adjusting for covariates. There are a number of different ways covariate-adjusted relative risks and risk differences can be estimated.

**Objectives:**

Our goal was to identify methods used to estimate covariate-adjusted relative risk and risk differences in RCTs published in high-impact journals with binary outcomes. Other secondary objectives included the identification of how covariates are chosen for adjustment and whether covariate adjustment results in an increase in statistical precision in practice.

**Methods:**

We included two-arm parallel RCTs published in *JAMA*, *NEJM*, *Lancet*, or the *BMJ* between January 1, 2018, and March 11, 2023, reporting relative risks or risk differences as a summary measure for a binary primary outcome. The search was conducted in Ovid-MEDLINE.

**Results:**

Of the 308 RCTs identified, around half (49%; 95% CI: 43–54%) reported a covariate-adjusted relative risk or risk difference. Of these, 82 reported an adjusted relative risk. When the reporting was clear (*n* = 65, 79%), the log-binomial model (used in 65% of studies; 95% CI: 52–76%) and modified Poisson (29%; 95% CI: 19–42%) were most commonly used. Of the 92 studies that reported an adjusted risk difference, when the reporting was clear (*n* = 56, 61%), the binomial model (used in 48% of studies; 95% CI: 35–62%) and marginal standardisation (21%; 95% CI: 12–35%) were the common approaches used.

**Conclusions:**

Approximately half of the RCTs report either a covariate-adjusted relative risk or risk difference. Many RCTs lack adequate details on the methods used to estimate covariate-adjusted effects. Of those that do report the approaches used, the binomial model, modified Poisson and to a lesser extent marginal standardisation are the approaches used.

**Supplementary Information:**

The online version contains supplementary material available at 10.1186/s13063-024-08690-w.

## Introduction

CONSORT guidelines recommend reporting relative and absolute measures of effect for binary outcomes in randomised controlled trials (RCTs), along with confidence intervals [1]. In tandem, the European Medicines Agency (EMA) and the US Food and Drug Administration (FDA) also recommend adjusting for covariates when estimating treatment effects to improve statistical efficiency [2, 3]. However, there are several choices to be made regarding which relative measure of treatment effect to report and how to adjust for covariates, which we consider in detail below.

### The advantages and disadvantages of different summary measures

When reporting relative measures of effect, the odds ratio is the most commonly reported summary measure [4–6]. Whilst a covariate-adjusted odds ratio can be readily estimated using logistic regression, some have argued that the odds ratio is not an intuitive summary measure [7, 8], and the magnitude of the intervention effect can be overstated if the odds ratios are misunderstood [9]. Furthermore, the odds ratio is non-collapsible, meaning that adjusting for covariates changes the meaning of the estimate—the unadjusted odds ratio targets the marginal estimand, while the adjusted odds ratio represents the conditional estimand [10]. In comparison, relative risks are easier to interpret, target the same estimand with and without covariate adjustment, and are less likely to be misinterpreted, meaning they can facilitate the translation of findings into practice [1, 8]. Estimation of both unadjusted and/or adjusted relative risks may therefore be of interest to many trials with binary primary outcomes.

### The benefits of covariate adjustment

Although more nuanced for binary outcomes, covariate adjustment is recommended for covariates used in any restricted randomisation [11, 12], and adjustment for other prognostic covariates can also increase statistical precision [2, 13, 14]. However, there is also evidence that adjustment for non-prognostic or weakly prognostic covariates can reduce precision [15–19]. Guidelines recommend that the covariates chosen for adjusted analysis or restricted randomisation should be predictive of the outcome and be pre-specified [3, 20–22]. The guidance also says the retrospective selection of covariates using data-driven approaches (e.g. testing for imbalance using statistical tests) should be avoided [3, 14].

### The challenges of estimation of adjusted relative risks and risk difference

Estimating an adjusted relative risk or risk difference can be more challenging than estimating an adjusted odds ratio, which can be implemented straightforwardly using logistic regression [23, 24]. For example, conventional estimators of a binomial regression model with a log or identity link can fail to converge, particularly in settings of low outcome event rates [8, 25, 26]. While other less-known approaches are available, they are not without their limitations. For example, modified Poisson can also yield fitted probabilities that are greater than one [26–28], and substitution approaches can underestimate standard errors [27]. More recently, an approach known as marginal standardisation, also known as G-computation or potential outcomes modelling, has seen some traction [29, 30]—but the implementation of this can be challenging for trial statisticians, although there are some new packages that can help with facilitation [31, 32].

### Overview of previous reviews of methods for estimating relative risks and risk differences

Despite guidance suggesting there is value in reporting covariate-adjusted relative risks and risk differences, reviews of RCTs with binary outcomes show many trials still do not follow this guidance [9, 33–37]. Firstly, binary outcomes are one of the most common outcome types, with the frequency of two-arm superiority RCTs having binary primary outcomes reported to range between 28 and 72% [33, 36, 37]. Among trials with a binary primary outcome, about 70% report an odds ratio, with much fewer reporting relative risks or risk differences [4, 5, 7, 9]. Secondly, covariate adjustment is performed in about a third of trials, possibly slightly less for binary outcomes and even less for relative risks or risk differences [9, 36]. Thirdly, whilst most covariates are chosen by pre-specification based on their prognostic relationship with outcomes [34, 35], most RCTs which use restricted randomisation do not adjust for all of the covariates used in the restriction [34, 38, 39].

Thus, whilst there is a growing appreciation for reporting adjusted relative risks and risk differences, there are undoubtedly barriers to implementation. None of the previous reviews have assessed what approaches are being used to estimate covariate-adjusted relative risks or risk differences. Thus, it is unclear how often different methods are applied in practice. There is also limited empirical evidence on the impact of covariate adjustment on statistical precision in practice—for example, if investigators are choosing non-prognostic covariates for adjustment, it might be the case that statistical precision decreases.

### Aims and objectives

This review was conducted with the primary aim of determining what methods are used to estimate unadjusted and adjusted relative risks and risk differences in two-arm parallel RCTs. We additionally sought to identify if it was possible to determine if the use of covariate adjustment results in increased statistical precision in practice. Using individually randomised trials published in a selection of high-impact journals, with a binary primary outcome, that report relative risks and risk differences, the objectives were to:

- Objective 1: Identify methods used to estimate unadjusted relative risks and risk differences (point estimates and confidence intervals) and the estimate proportion of RCTs that use such methods.
- Objective 2: Estimate the proportion of RCTs that report a covariate-adjusted relative risk or risk differences (along with confidence intervals, standard errors, and *p*-values) within a primary, secondary, or exploratory analysis; along with the proportion that adjust for covariates used in any restricted randomisation.
- Objective 3: Identify methods used to estimate the covariate-adjusted relative risks and risk differences (point estimates and confidence intervals) and estimate the proportion of RCTs that use such methods.
- Objective 4: Describe how covariates are chosen for adjustment, including pre-specification of clinically important variables or data-driven approaches.
- Objective 5: Compare the unadjusted and adjusted point estimates, standard errors, and *p*-values.

## Methods

Since this review focuses on RCT methodology, it was not eligible for registration on PROSPERO. However, the protocol was developed using Preferred Reporting Items for Systematic Reviews Meta-Analyses (PRISMA) guidelines [40] and is available in Additional file 1 (Table A1).

### Eligibility criteria

We included two-arm, individually randomised trials published in four selected high-ranking journals that publish across diverse clinical fields: the *Journal of the American Medical Association* (JAMA), the *New England Journal of Medicine* (NEJM), the *Lancet*, and the *BMJ*. These journals were chosen because adherence to CONSORT recommendations should be higher within these journals, and therefore, reporting of relative risks and risk differences as a summary measure for a binary primary outcome should be more frequent than in lower-impact journals. The dates of the search were limited to January 1, 2018, and March 11, 2023, to align with our sample size justification (see below). The review is organised into two parts. The first part, referred to as the overall review sample, addresses the first two objectives (estimation of unadjusted effects and proportion of RCTs that report covariate-adjusted effects), and the second part, referred to as the “nested review,” addresses the other three objectives (estimation of adjusted effects).

#### Inclusion criteria

##### Overall review sample

- i. RCTs (superiority and non-inferiority) that were published in either the JAMA, NEJM, Lancet, or BMJ between January 1, 2018, and March 11, 2023, with a binary primary outcome and reporting a (unadjusted or covariate-adjusted) relative risk or risk difference.

##### Nested review sample

- ii. The sub-sample of RCTs identified from the overall review sample reporting a covariate-adjusted relative risk or risk difference.

#### Exclusion criteria

##### Overall review sample

i. All non-human, vaccine, or drug safety trials.
ii. Cluster or cross-over randomised studies, pilot, feasibility, phase I or II trials, trials with more than two arms, and factorial trials.
iii. RCTs that are not primary reports but secondary publications (e.g. follow-up reports) and publications primarily reporting health economic analyses.
iv. RCTs that only report proportions (i.e. no treatment effect), odds ratios, or other summary measures of effect that are not relative risks or risk differences.
v. RCTs that evaluate co-primary outcomes or multiple primary outcomes. This includes studies that concurrently conducted two or more trials and reported the outcomes in one article.
vi. RCTs that have non-binary primary outcomes (e.g. continuous, rate, ordinal, count, or time-to-event outcomes).
vii. Duplicate publications, abstracts, ongoing studies, conference proceedings, research letters, commentaries, editorials, or review articles.

##### Nested review sample

i. RCTs that do not report any values of either the point estimate, confidence interval or a *p*-value for the covariate-adjusted relative risk or risk difference within the main report for the primary, secondary, or exploratory analysis.

### Sample size justification

We determined the sample size based on a combination of a size that would be feasible and allow us to estimate various quantitative objectives with reasonable precision. For the overall review sample, we aimed to identify about 300 studies that met our inclusion criteria (i.e., two-arm RCTs with a binary primary outcome reporting relative risks or risk differences as a summary measure). Assuming approximately 30% of RCTs implement covariate adjustment [34], a sample size of 300 would allow us to estimate the percentage of studies implementing covariate adjustment, with a 95% CI of approximately 25% to 35% (objective 2). A sample size of 300 would also allow us to estimate the proportion of trials using one of the more common approaches to estimate unadjusted effects (e.g. modified Poisson) with similar precision. Furthermore, identifying 100 studies from the initial sample of 300 that implement covariate adjustment will be of reasonable size to allow us to summarise the methods used to estimate covariate-adjusted relative risks and risk differences, and how covariates are selected for adjustment (to evaluate objectives 3 and 4) and quantitatively compare the unadjusted and covariate-adjusted results (objective 5).

To identify about 300 studies that meet these broad inclusion criteria, we anticipated we needed to screen about 900 RCT abstracts since about one-third of RCTs report a binary primary outcome and report either a relative risk or risk difference [36]. It was estimated that approximately 245 RCTs are published each year in the four target journals (estimated via a scoping search). Thus, to achieve a target of about 900 RCTs, it was estimated that the search should cover the period from 2018 to 2023, roughly identifying 980 RCTs.

### Search strategy

A combination of relevant keywords and Boolean operators were used to search Ovid Medline. Search strings were developed using recommended filters [41]. See Table A2 for details of the search strategy.

### Screening and selection

The records identified were imported into a bibliographic referencing software programme [42]. One reviewer (JT) independently screened the titles and abstracts for inclusion in the overall review sample using the Covidence systematic review software [43]. Those RCTs identified as meeting the eligibility criteria for the overall review sample were then further screened (again by one reviewer, JT) for inclusion in the nested review sample. Details are outlined below.

#### Overall review sample screening

The initial process involved screening the title and abstracts of the identified RCTs (anticipated to be around 980 reports) to identify whether relative risks or risk differences were reported as a summary measure for the primary outcome. Our working definition of the primary outcome used the following hierarchy. First and foremost, records were searched for any clear definition of a primary outcome in the manuscript. When the primary outcome was not clearly specified, we chose the outcome used for sample size calculation, and where that was not clear, the first outcome was reported in the abstract. For those RCTs that meet the eligibility criteria of having a single binary primary outcome, all full texts were retrieved to ascertain if a relative risk or risk difference was reported. Those that met all these criteria were included in the overall review sample.

### Nested review sample screening

For those RCTs that met the eligibility criteria of the overall review sample, all full texts (anticipated to be around 300 reports) were retrieved to ascertain if a covariate-adjusted relative risk or risk difference was reported for the primary outcome (either for the primary analysis, a secondary or sensitivity analysis).

### Data extraction

#### Overall review sample

The following information was extracted from each study:

1. Information about the authors, year of publication, the journal, whether the trial was a multi-centre or single-centre study, and the sample size (total number of participants randomised).
2. The type of unadjusted summary measures reported (i.e. relative risk and/or risk difference) for the primary binary outcome.
3. The analysis method used to obtain the estimate of relative risk and/or risk difference and its corresponding confidence interval.
4. Whether a restricted method of randomisation was used, and where a restricted method was used how many covariates were included in the restriction.
5. Whether a covariate-adjusted relative risk or risk difference is reported and identify if it is reported for the primary, secondary, or exploratory analysis.

#### Nested review sample

A more detailed extraction was then undertaken again using the full texts of the anticipated 100 trials that met the eligibility criteria for the nested review sample (i.e. two-arm RCTs with a binary primary outcome that reports a covariate-adjusted relative risk or risk difference). The additional information extracted was:

1. When reported, the method used to estimate the covariate-adjusted relative risk or risk difference.
2. Whether the covariate-adjusted analysis was fully or partially adjusted for covariates used in the randomisation and whether the analyses were adjusted for any additional covariates (and if so, how many).
3. When reported, how covariates were chosen for adjustment, including selection based on the pre-specification of clinically important variables, those used in the randomisation, or data-driven approaches.
4. The values of the point estimate and measures of uncertainty (confidence interval, standard error, and *p*-value) for the unadjusted and adjusted relative risk or risk difference.

In the setting where more than one covariate-adjusted relative risk or risk difference was reported, we extracted this information for the one which was the most comprehensive covariate-adjusted model. The methods for estimation of a covariate-adjusted relative risk or risk difference were categorised as follows:

1. Binomial model (or log-binomial)—generalised linear model with binomial distribution and log link (for estimation of relative risk) or identity link (for estimation of risk difference).
2. Modified Poisson—generalised linear model with Poisson distribution, robust standard errors, and log link (for estimation of relative risk) or identity link (for estimation of risk difference).
3. Marginal standardisation—generalised linear model with binomial distribution and logit link; then predict outcomes and average across groups, with log link (for estimation of relative risk) or identity link (for estimation of risk difference).
4. Linear model—linear model with Gaussian distribution, robust standard errors with identity link (for estimation of risk difference only).

These categorisations were used as initial scoping work suggested these were the more commonly used approaches (see later text for assumptions made when categorising here). Approaches that did not clearly align with one of these were classified as ‘other’. In addition, more detailed information was extracted (verbatim text of the approach used) and reported in supplementary tables. When these data were not clearly reported in the main report, the supplementary materials (e.g. protocols and statistical analysis plans) were also accessed for further details. We used these same classifications for the estimation of the unadjusted effects but note that an unadjusted point estimate is simply the ratio or difference and so it was not expected that this trivial detail would be reported, where confidence intervals were reported, we were seeking to identify the method that primarily related to how this had been derived.

Data was extracted using a standardised data extraction proforma developed in AirTable. The proforma underwent iterative review and feasibility testing by the project team to address discrepancies and ensure consistency (see Table A3). In a pilot/training phase, two reviewers (JT and LM) independently performed the data extraction for the first 21 studies and then resolved all discrepancies by discussion. Subsequent to this, the remaining data was extracted by one reviewer (JT), with consultation with the wider team in cases of uncertainty.

#### Methodological assumptions

Several assumptions were made about the methods used to estimate the relative risk or risk difference when the reporting was not clear or comprehensive (for reporting in main tables, though verbatim text is provided in supplementary tables). When it was reported that a generalised linear model was used, but without information on the link or distribution, it was assumed that this was the log-binomial model for the relative risk and the identity link for the risk difference. Likewise, generalised linear mixed models and generalised estimating equations are categorised as the log-binomial model (for estimation of relative risk) or binomial-identity model (for estimation of risk difference). When authors proposed an alternative approach to handle non-convergence, e.g. modified Poisson for the log-binomial model, we report the method that was implemented and reported. When authors report using “G-computation”, logistic regression with delta standard errors or bootstrapping, or other descriptions of standardisation (such as the marginal average of predicted values from the logistic regression), these were classified as marginal standardisation. Modified Poisson using generalised estimating equations is categorised as modified Poisson.

The approach was classified as unclear when the authors reported using mixed-effect modelling with an exchangeable correlation but with no further detail. Similarly, when authors reported the use of logistic regression to estimate a relative risk or risk difference without any other detail, this is categorised as unclear. When authors reported using an approach that provides a statistical test, such as the Cochran-Mantel–Haenszel test, chi-square or Fisher’s exact test, Wald likelihood ratio approximation tests or *Z*-test, without any clear information on how the confidence intervals were estimated, this was also categorised as unclear.

### Data analysis

The number of studies identified, screened, and excluded were reported, and the findings are presented in a PRISMA flowchart. A descriptive summary of the trials included in the review is provided, summarising using numbers (and percentages) for categorical data or medians (and interquartile ranges) for continuous data. The reliability between the data extractions performed in duplicate is reported using percentage agreement and unweighted Kappa statistics, which measures the agreement between two raters classifying categorical items [44]. When describing numbers and percentages related to the key objectives, such as proportions of analyses using the different candidate approaches, numbers and percentages were reported, and 95% confidence intervals around these percentages were reported using the Wilson score confidence interval method [45]. To compare precision between the unadjusted and adjusted effects (where few trials were anticipated to report standard errors) we estimate the standard error by subtracting the upper confidence interval from the lower confidence interval and dividing by 3.92 (working on the log scale for relative risks).

All descriptive, exploratory analyses and data visualisations were performed using R Statistical Software version 4.4.1 [46]. Kappa statistics were estimated using the IRR package [44]. The 95% CIs were computed using the Wilson score confidence intervals in the gt_summary R package [45].

## Results of the systematic review

### Identification of eligible studies

**Fig. 1:**
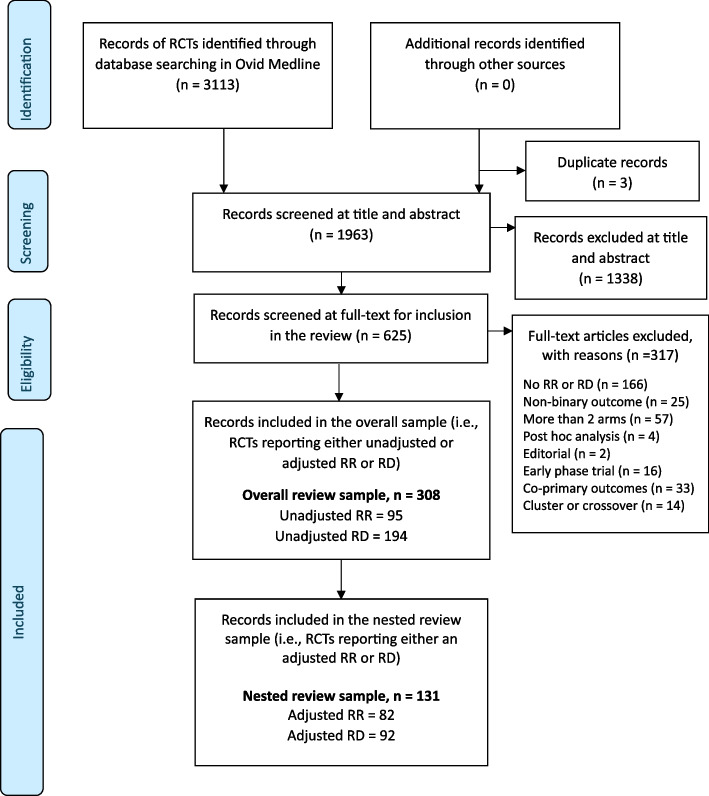
PRISMA flow diagram of the selection process for eligible studies. The overall review sample includes RCTs published between January 1, 2018, and March 11, 2023, in selected high-impact journals, with a primary binary outcome, that report either a relative risk (RR) or risk difference (RD) (unadjusted or adjusted) as a summary measure. The numbers reported for unadjusted RR and RD are not mutually exclusive to the reporting of adjusted RR and RD. RR, relative risk; RD, risk difference. The nested review sample is a sub-sample of RCTs identified from the overall review sample that reports a covariate-adjusted RR or RD for the primary, secondary, or exploratory analysis. The numbers reported for adjusted RR and RD are not mutually exclusive

Of the 3113 records that were identified and screened for relevance at the title and abstract stage, three were excluded as duplicates and 1338 were excluded on an abstract screen (Fig. 1). Then, 1963 full-text articles were screened for inclusion against the eligibility criteria, and 308 studies met the criteria required for the overall review sample (see flow chart for details of reasons why studies were excluded, mostly (*n* = 191) due to not reporting a relative risk or risk difference). These 308 studies were RCTs that reported either an unadjusted or covariate-adjusted relative risk, risk difference or both. Of these 308 studies, 131 were identified to report either a covariate-adjusted relative risk, risk difference, or both and were eligible for inclusion in the nested review sample. None of the included studies reported on more than one RCT in each trial report.

### Agreement across data extraction

The percentage agreement across the two independent data extractions for the 21 studies that were extracted in duplicate was 92.8%, and unweighted Kappa was 0.9 (95% CI: 0.80–0.99). Most discrepancies were due to a lack of clarity in the description provided by the authors regarding the methods used to derive summary measures for the unadjusted analysis (Table A4). In many cases, it was unclear how the confidence intervals were derived. Other discrepancies were due to a lack of clarity in approach when both the relative risk and risk difference were reported. It was also commonly unclear whether the *p*-value reported was for the relative risk or risk difference.

### Characteristics of included studies

Of the 308 RCTs in the overall review sample, most trials were published in JAMA (108/308, 35%) or NEJM (108/308, 35%) compared to Lancet (75/308, 24%) or BMJ (17/308, 6%) (Table 1). The studies were all published between 2018 and 2023. Most (293/308, 95%) were multi-centre studies with an average sample size in the control and intervention arms of 357 (IQR: 158–717) and 367 (IQR: 176–729), respectively. Using a restricted method of randomisation was common (213/308, 69%), with the average number of covariates used in the randomisation reported to be 2 (IQR: 1–3). Of those that used restricted randomisation (*n* = 213), about half (107, 50%; 95% CI: 43–57%) reported adjusting for all those covariates used in the restriction; whilst an additional 18 (9%; 95% CI: 5%, 13%) partially adjusted for some of the covariates; in about a fifth (45, 21%; 95% CI: 16–27%) the analyses did not adjust for these covariates and in the remainder (43, 20%; 95% 15–26%), it was unclear. Of the 308 RCTs, around half (150/308, 49%; 95% CI: 43–54%) reported a covariate-adjusted relative risk or risk difference.

**Table 1:** Characteristics of studies included in the overall review sample

	**Trials that reported either unadjusted or adjusted RR or RD** ***N* = 308**
**Characteristics of the included studies**	
Journal (*N*, %)	
*JAMA*	108/308 (35%)
*NEJM*	108/308 (35%)
*Lancet*	75/308 (24%)
*BMJ*	17/308 (6%)
Publication year (*N*, %)	
*2018*	60/308 (19%)
*2019*	71/308 (23%)
*2020*	53/308 (17%)
*2021*	54/308 (18%)
*2022*	58/308 (19%)
*2023*	12/308 (4%)
Type of centre (*N*, %)	
*Multi-centre*	293/308 (95%)
No. of participants, median [IQR]^a^	
*Intervention arm*	367 [176, 729]
*Control arm*	357 [158, 717]
**Practices relating to covariate adjustment (*N*/denominator, %, 95% CI)**	
Used a restricted randomisation (*N*, %)	213/308, 69%
*Number of randomisation covariates, median [IQR]*	2 [1.0, 3.0]
*Complete adjustment for randomisation covariates (N, %)*	107/213, 50% (95% CI: 43%, 57%)
*Partial adjustment for randomisation covariates (N, %)*	18/213, 8.5% (95% CI: 5.2%, 13%)
*No adjustment for randomisation covariates (N, %)*	45/213, 21% (95% CI: 16%, 27%)
*Unclear adjustment for randomisation covariates (N, %)*	43/213, 20% (95% CI: 15%, 26%)
Report adjusted RR or RD^b^ (*N*, %)	150/308, 49% (95% CI: 43%, 54%)

*RR* relative risk, *RD* risk difference, *N* number, *IQR* interquartile range, *CI* confidence interval, *%* percent, *JAMA* Journal of the American Medical Association, *NEJM* New England Journal of Medicine, *BMJ* British Medical Journal

^a^This is the number of participants randomised

^b^Report of adjusted or unadjusted RR or RD at any point in the report

### Methods for estimating unadjusted relative risks and risk differences

#### Unadjusted relative risks

Of the 308 studies, 95 reported an unadjusted relative risk (Table 2). In the majority (*n* = 65, 68%; 95% CI: 58–77%) of these reports, the method used to estimate the confidence intervals for the unadjusted relative risk was unclear. Of the 30 RCTs that reported an unadjusted relative risk and where the reporting of the method for the confidence interval was clear, the most common method used was the log-binomial model, used in 21 (70%; 95% CI: 50–85%) of the studies; followed by modified Poisson, used in 7 (23%; 95% CI: 11–43%) of the studies; and only in one (3%; 95% CI: 0–19%) study was marginal standardisation reported to be used. See Table A5 for a granular description of the methods identified. Of the 95 studies that reported an unadjusted relative risk, almost all studies reported point estimates and upper and lower confidence intervals. However, whilst *p*-values for the unadjusted relative risk were reported for most studies (77, 81%), they were not always reported. No studies reported standard errors of effects.

#### Unadjusted risk differences

Of the 308 studies, 194 RCTs reported an unadjusted risk difference. In the majority (*n* = 139, 72%; 95% CI: 65–78%) of these 194 reports, the method used to estimate the confidence intervals for the unadjusted risk difference was unclear (Table 2). Of the 55 RCTs that reported an unadjusted risk difference and where the reporting of the confidence interval was clear we did not identify a universally more common approach: the binomial model was used in 9 (16%; 95% CI: 8–29%) studies; the linear model used in 6 (11%; 95% CI: 4.5–23%) studies; marginal standardisation used in 4 (7%; 95% CI: 2–18%) studies; and modified Poisson used in 2 (4%; 95% CI: 1–14%) studies. Moreover, we identified that many other, different, approaches were used as in 34 (62%; 95% CI: 48–74%) of the studies, the approach was classified as ‘other’. See Table A6 for a granular description of the methods identified. Again, of the 194 RCTs that reported an unadjusted risk difference, most reported point estimates and upper and lower confidence intervals. However, only around half of the studies, 127/194 (65%), reported *p*-values for the unadjusted risk difference; and no studies reported standard errors of estimated effects.

**Table 2:** Methods used for estimating unadjusted and adjusted relative risks and risk differences and completeness of reporting

	**Trials that reported either unadjusted or adjusted RR or RD** ***N* = 308**			
	**Trials that reported either unadjusted or adjusted RR** ***N* = 137**		**Trials that reported either unadjusted or adjusted RD** ***N* = 244**	
	**Unadjusted RR** ***N* = 95**	**Adjusted RR** ***N* = 82**	**Unadjusted RD** ***N* = 194**	**Adjusted RD** ***N* = 92**
**Method used (*N*/denominator, %, 95% CI)**				
*Unclear*^a^	65/95, 68% (58%, 77%)	17/82, 21% (13%, 31%)	139/194, 72% (65%, 78%)	36/92, 39% (29%, 50%)
*Of those that report a clear approach*				
*Binomial model*^b,c^	21/30, 70% (50%, 85%)	42/65, 65% (52%, 76%)	9/55, 16% (8%, 29%)	27/56, 48% (35%, 62%)
*Marginal standardisation*	1/30, 3% (< 1%, 19%)	2/65, 3% (< 1%, 12%)	4/55, 7% (2%, 18%)	12/56, 21% (12%, 35%)
*Modified Poisson*^d^	7/30, 23% (11%, 43%)	19/65, 29% (19%, 42%)	2/55, 34% (< 1%, 14%)	4/56, 7% (2%, 18%)
*Linear model*	0 (0%)	0/65 (0%)	6/55, 11% (5%, 23%)	6/56, 11% (4%, 23%)
*Other*	1/30, 3% (< 1%, 19%)	2/65, 3% (< 1%, 12%)	34/55, 62% (48%, 74%)	7/56, 13% (6%, 25%)
**Completeness of reporting of point estimates and measures of uncertainty (*N*/denominator)**				
*Point estimate*	95/95 (100%)	82/82 (100%)	194/194 (100%)	91/92 (99%)
*LCI*	95/95 (100%)	82/82 (100%)	184/194 (95%)	88/92 (96%)
*UCI*	94/95 (99%)	81/82 (99%)	179/194 (92%)	87/92 (95%)
*P-value*^d^	77/95 (81%)	66/82 (80%)	127/194 (65%)	53/92 (58%)
*SE*	0 (0%)	0 (0%)	0 (0%)	0 (0%)

*RR* relative risk, *RD* risk difference, *CI* confidence interval, *N* number, *LCI* lower confidence interval, *UCI* upper confidence interval, *SE* standard error

^a^Unclear represents situations where the information was unclear, not reported, unable to be determined, or missing. See text for details

^b^Binomial model with log link for RR and identity link for RD

^c^In one RCT, the RR and 95% CI were estimated via the log-binomial model and *P*-value from the Cochran-Mantel–Haenszel test

^d^In one study, the authors only reported adjusted *p*-values estimated using modified Poisson. It is assumed that the adjustment for centre as a fixed and random effect is a covariate-adjusted effect. For the RD, the modified Poisson is used with the identity link

### Methods used to estimate the covariate-adjusted treatment effects

#### Adjusted relative risks

Of the 308 studies, 82 reported an adjusted relative risk (Table 3). In about a quarter of these reports, the method used was unclear, 17 (21%; 95% CI: 13–31%). When the reporting was clear, the log-binomial model was the most common method used in 42 (65%; 95% CI: 52–76%) studies; then modified Poisson which was used in 19 (29%; 95% CI: 19–42%); and marginal standardisation used in two (3%; 95% CI: < 1–12%) studies. See Table A5 for a granular description of the methods identified. Most studies (82, 100%) reported point estimates, and upper and lower confidence intervals (82, 99%). However, *p*-values for the adjusted relative risk were reported less frequently (reported in 66 (80%) studies); and no studies reported standard errors.

#### Adjusted risk differences

Of the 308 studies, 92 reported an adjusted risk difference. The method used was unclear in a considerable proportion, 36 (39%; 95% CI: 29–50%). When the reporting was clear, the binomial model, used in 27 (48%; 95% CI: 35–62%) studies, was the most common method. Then marginal standardisation was used in 12 (21%; 95% CI: 12–35%) studies; then linear model which was used in 6 (11%; 95% CI: 4–23%) studies; and modified Poisson used in 4 (7%; 95% CI: 2–18%) studies. However, a small proportion of methods in 7 (13%; 95% CI: 6–25%) were classified as other. Most studies (91, 99%) reported point estimates, upper confidence intervals (88, 96%) and lower confidence intervals (87, 95%). However, *p*-values for the adjusted risk difference were reported less frequently (reported in 53 (58%) studies); and no studies reported standard errors. See Table A6 for a granular description of the methods identified.

### Whether covariate-adjusted effects are reported for primary analyses

Of the studies included in the nested review sample, 82 studies reported a covariate-adjusted relative risk, and 92 reported a covariate-adjusted risk difference (Table 3). Of the 82 studies that reported an adjusted relative risk, in 67 (82%; 95% CI: 71–89%) studies, the adjusted relative risk was reported as the primary analysis; in 5 (6%; 95% CI: 2–14%) studies this was for a secondary analysis; and in 10 (12%; 95% CI: 6–22%) studies this was for an exploratory or sensitivity analysis. Similarly, of the 92 studies that reported a covariate-adjusted risk difference, in 74 (80%; 95% CI: 71–88%) studies this was for the primary analysis; in 6 (7%; 95% CI: 3–14%) studies this was for a secondary analysis; and in 12 (12%; 95% CI: 7–22%) studies this was for an exploratory or sensitivity analysis.

**Table 3:** Current practice related to covariate-adjusted analysis in the nested review sample

	**Trials that reported either adjusted RR or RD** ***N* = 131**	
	**Trials that report adjusted RR** ***N* = 82**	**Trials that report adjusted RD** ***N* = 92**
Covariate-adjusted analysis reported for the*: (*N*/denominator, %, 95% CI)		
*Primary analysis*	67/82, 82% (71%, 89%)	74/92, 80% (71%, 88%)
*Secondary analysis*	5/82, 6% (2%, 14%)	6/92, 7% (3%, 14%)
*Sensitivity or exploratory analysis*	10/82, 12% (6%, 22%)	12/92, 13% (7%, 22%)
*Unclear*^*1*^	0/82 (0%)	0/92 (0%)
Justification for covariate adjustment (*N*, %)		
*Pre­specification*^a^	73/82, 89% (80%, 95%)	84/92, 91% (83%, 96%)
*Data-driven—*post hoc	4/82, 5% (2%, 13%)	2/92, 2% (< 1%, 8%)
*Unclear*^b^	5/82, 6% (2%, 14%)	6/92, 7% (3%, 14%)
Additional covariates included in adjustment^c^ (*N*, %)	34/82, 41% (31%, 53%)	33/92, 36% (26%, 47%)
*Number of additional covariates, Median (IQR)*	3 [2.0, 5.0]	3 [2.00, 4.00]

*RR* relative risk, *RD* risk difference, *IQR* interquartile range, *CI* confidence interval, *N* number. *%* percent

^a^Pre­specification includes covariates used in the randomisation or perceived prognostic importance. Data-driven approaches include post hoc analysis due to a lack of balance or statistical significance

^b^Unclear represents situations where the information was unclear, not reported, unable to be determined, or missing. See text for details

^c^Additional covariates refer to adjustment in addition to those covariates used in the randomisation

### Justification of the choice of covariates used for adjustment

For the 82 studies that reported an adjusted relative risk, in the vast majority (73, 89%; 95% CI: 80–95%) of these 82 studies, the covariates used in the adjustment were pre-specified (which included randomisation covariates). In only a few (4, 5%; 95% CI: 2–13%) studies, covariate adjustment resulted from data-driven post-hoc analyses or unclear approaches (5, 6%; 95% CI: 2–14%). In addition, 34 (41%; 95% CI: 31–53%) studies reported adjusting for covariates other than those included in a restricted randomisation. The average number of additional covariates (i.e. in addition to those adjusted because they were used in the randomisation) used in these studies was 3 (IQR: 2–5).

Of the 92 studies that reported an adjusted risk difference, a restricted method of randomisation was used in 85 (92%; 95% CI: 84–97%) studies. For these studies, the reported rationale for choosing these covariates was mostly pre-specification in 84 (91%; 95% CI: 83–96%); rather than data-driven in 2 (2%; 95% CI: < 1–8.4%) or unclear approaches in 6 (7%; (95% CI: 3–14%). In addition, 33 (36%; 95% CI: 26–47%) studies reported adjusting for covariates other than those included in a restricted randomisation. The average number of additional covariates used in these studies was 3 (IQR: 2–4).

### Comparison of adjusted and unadjusted effects

#### Relative risks

**Fig. 2:**
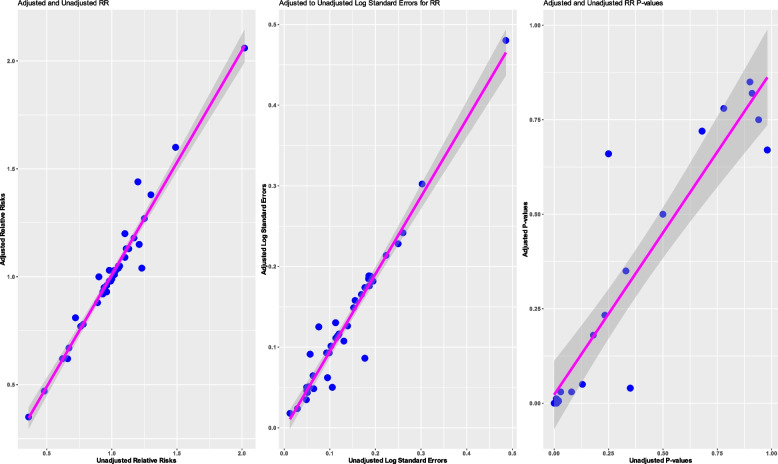
Comparison of adjusted to unadjusted relative risks. Footnotes: relative risk; CIs, confidence intervals. If the point falls below the line of equality, then the value is smaller under the adjusted analysis than under the unadjusted analysis (as anticipated per theory). However, if the point is above the line of equality, then the value is larger under the adjusted analysis than under the unadjusted analysis

Of the 82 studies that reported covariate-adjusted relative risk, 41 reported both an unadjusted and adjusted relative risk (Fig. 2). The adjusted and unadjusted point estimates were mostly observed to be similar for each study, with occasional larger differences that went in both directions (sometimes the adjusted relative risk was larger than the unadjusted relative risk and vice versa). A similar pattern was observed for standard errors and *p*-values: mostly these were similar between the adjusted and unadjusted analyses, but sometimes there were larger differences, and these differences went in both directions—so that sometimes the standard error from an adjusted analysis could be smaller than from the unadjusted analysis and sometimes larger.

#### Risk differences

**Fig. 3:**
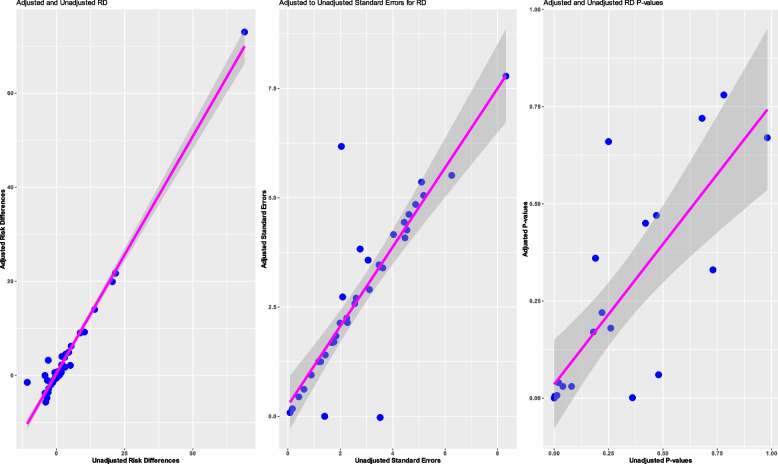
Comparison of adjusted to unadjusted risk differences. Footnotes: RD, risk difference; CIs, confidence intervals. If the point falls below the line of equality, then the value is smaller under the adjusted analysis than under the unadjusted analysis (as anticipated per theory). However, if the point is above the line of equality, then the value is larger under the adjusted analysis than under the unadjusted analysis

Of the 92 studies that reported covariate-adjusted risk differences, 42 reported both an unadjusted and adjusted risk difference. The reader is referred to in Fig. 3, and the findings are similar to those of the relative risk.

## Discussion

### Summary of findings

Only around half of RCTs (trials published in high-impact journals with a binary primary outcome that uses a risk difference or relative risk for summarising the impact of the treatment) report a covariate-adjusted relative risk or risk difference. This is despite the fact that substantially more of the RCTs included in the review used a restricted randomisation procedure, and the increase in statistical precision that has been reported to result arise when adjusting for prognostic covariates. However, in our pairwise comparison of adjusted vs. unadjusted relative risks and risk differences, we did not find evidence that covariate adjustment universally improves statistical precision.

Those trials that do report covariate-adjusted relative risks or risk differences mostly pre-specify the covariates for adjustment and adjust for covariates included in any restricted randomisation. When it comes to reporting unadjusted relative risks and risk differences, most do not clearly report the approach used (this is despite the fact that using something like Fisher’s exact test only provides a *p*-value and not a confidence interval); and of those that do report the approach, mostly use a binomial model (with log or identity link respectively) or modified Poisson.

When it comes to reporting adjusted relative risks and risk differences, a significant number (estimated to be in the region of 40% for adjusted risk differences) still do not report the approach used. Of those that do report the approach used, again, the binomial model is a common approach, and whilst modified Poisson is commonly used to estimate an adjusted relative risk, marginal standardisation is commonly used to estimate an adjusted risk difference. For the relatively small number of trials that reported both an adjusted relative risk and unadjusted relative risk; or adjusted risk difference and unadjusted risk difference, we observed that statistical precision did not always increase on adjustment—with an increase or decrease on adjustment occurring roughly an equal amount of times.

#### Comparison with the broader literature

A number of reviews have examined methods for estimating summary measures for binary outcomes over the last decade, but none of these reviews focused on relative risks and risk differences [33–37]. These reviews have identified that most trials adjust for covariates used during randomisation [34]. However, only about half of the trials included in this review reported a covariate-adjusted relative risk or risk difference, whereas 70% of them used a restricted randomisation. Our findings mostly concur with those from other reviews, where it has been identified that the practice of covariate adjustment is mostly guided by covariates included in restricted randomisation [1, 12, 34, 47]; but that a significant proportion of trials do not report a covariate-adjusted treatment effect for their primary analysis [35, 38].

### Limitations of this review

This review included only two-arm RCTs published in one of four high-impact journals with a binary primary outcome reporting either a relative risk or risk difference. We focused on high-impact journals as these journals are likely to contain more information about the methods used—the focus of our review. However, as such, this very likely means our findings are not representative of all RCTs. Only trials which specifically reported either a relative risk of risk difference were included, as the primary focus was on methods used to estimate these summary measures, but this means this work cannot make inferences about the proportion of trials that report a covariate-adjusted treatment effect for example (this has not captured those trials that report a covariate-adjusted odds ratio). There are also more nuanced details that we did not report on. For example, some trials reported using an alternative approach when the primary method did not converge. Neither did we elicit very nuanced details on the approach used, for example, the method of optimisation or details on the methods used to estimate standard errors. Studies evaluating co-primary outcomes were not considered since they raise complex issues relating to type I and type II error control in sample size calculation and final analysis [48].

Double extraction was performed with an independent reviewer for approximately 10% of the papers, which showed good agreement (93%) on the core items of interest. However, we did not perform independent and duplicate data extraction for the entire sample. Furthermore, due to time and resource constraints, study authors were not contacted to resolve any issues around unclear reporting. Likewise, we did not compare the pre-specified covariates reported in the main report with those of the statistical analysis plan; therefore, it is assumed this has not changed. Moreover, whilst one of our objectives was to compare the estimated unadjusted and adjusted relative risks and risk differences—to identify if, in practice, adjusted is resulting in an increase in statistical precision, we only had a small sample of studies that reported both metrics and so here our findings are limited to a small sample.

### Implications for practice

There are valid reasons for estimating covariate-adjusted relative risks and risk differences when reporting findings from randomised trials. There are methods available to facilitate the estimation of these summary metrics, yet even in high-impact journals, there is both a lack of clarity around approaches used for estimation and a lack of uptake in the use of methods that are available.

#### Added value of the review

This systematic review is one of the most comprehensive reviews cataloguing methods for estimating relative risks and risk differences used in current practice. Other reviews have identified that about one third of RCTs report covariate-adjusted treatment effects (by the nature of these reviews, these treatment effects will often be the odds ratio [4, 6, 8, 24, 33, 36, 49, 50], whereas we identified that about half of RCTs with a binary outcome that report either a relative risk or risk difference report a covariate-adjusted treatment effect. One possible explanation for this difference might be that those trials that report a relative risk or risk difference are those that are more conscious about following reporting guidance. In part, this might be hypothesised to be explained by how more complicated methods can be required to implement covariate-adjusted for the estimation of relative risks and risk differences (e.g. using marginal standardisation or modified Poisson). Yet, we identified that some methods that might be considered more novel (such as modified Poisson and marginal standardisation) are used quite frequently. We identified a significant lack of clarity in reporting how unadjusted relative risk and risk differences are estimated.

## Conclusions

Numerous methods are currently being used in practice to estimate relative risks and risk differences, indicating that the availability of methods is perhaps no longer a barrier; rather, guidance on which methods are best is what is lacking. The reporting of methods for estimating unadjusted relative risk or risk difference is still inadequate in some areas. Furthermore, the reporting of unadjusted and adjusted risk treatment effects is still low, but the rationale for covariate adjustment was commonly reported.

**Table:** 

**What is already known?** ➢ There are numerous methods available for estimating relative risks and risk differences. ➢ Covariate adjustment is recommended to improve precision and power.
**What is new?** ➢ Around half of trials published in the top high-impact journals with a binary primary outcome that report a relative risk or risk difference do not report a covariate-adjusted relative risk or risk difference. ➢ Those that do report a covariate-adjusted relative risk or risk difference mostly choose their covariates in advance and adjust for covariates used in the randomisation. ➢ When reporting unadjusted relative risks and risk differences, most trials do not provide adequate information on the method used. ➢ When reporting covariate-adjusted relative risks and risk differences, more trials report details on the approach used—but there is still a significant lack of reporting. ➢ Of those trials that do clearly report how they estimate covariate-adjusted relative risks and risk differences, common approaches include the binomial model, modified Poisson and marginal standardisation.

## Supplementary Information

Supplementary Material 1: Table A2: Summary of the search strategy and papers identified via Ovid Medline. Table A3: Data Dictionary Codebook for review of binary outcomes. Table A4: Discrepancies and consensus agreement. Table A5: Detailed description of methods for estimating relative risks from overall and nested sample. Table A6: Detailed description of methods for estimating risk differences from overall and nested sample.

## Abbreviations

BMJ: *British Medical Journal*
CI: Confidence interval
CONSORT: Consolidated Standards of Reporting Trials
EMA: European Medicines Agency
FDA: US Food and Drug Administration
GEE: Generalised estimating equations
GLM: Generalised linear models
GLMM: Generalised linear mixed models
HC1: Huber White Sandwich Estimator
IQR: Interquartile range
JAMA: *Journal of the American Medical Association*
LM: Linear model
NEJM: *New England Journal of Medicine*
OR: Odds ratio
RCTs: Randomised controlled trials
RD: Risk difference
RR: Relative risk
SE: Standard error
tMLE: Targeted Maximum Likelihood Estimator Statistical Analysis Plans

## References

[1] Moher D, Hopewell S, Schulz KF, Montori V, Gotzsche PC, Devereaux PJ, et al. CONSORT 2010 Explanation and Elaboration: updated guidelines for reporting parallel group randomised trials. BMJ. 2010;340(mar23 1):c869-c. 10.1136/bmj.c869 PMC2844943 20332511

[2] Office of the Federal Register NAaRA. Adjusting for Covariates in Randomized Clinical Trials for Drugs and Biological Products; Draft Guidance for Industry; Availability. [Government]. In: 27627 F, editor. USA: Office of the Federal Register, National Archives and Records Administration 2021. p. 27627–9.

[3] EMA. Guideline on adjustment for baseline covariates in clinical trials In: (CHMP) CfMPfHU, editor. London: EMA; 2015. p. 1–11.

[4] Ackerman B, Lesko CR, Siddique J, Susukida R, Stuart EA. Generalizing randomized trial findings to a target population using complex survey population data. Stat Med. 2021;40(5):1101–20. 33241607 10.1002/sim.8822 PMC8034867

[5] Stang A, Rothman KJ. Statistical inference and effect measures in abstracts of randomized controlled trials, 1975–2021. A systematic review. Eur J Epidemiol. 2023;38(10):1035–42. 37715928 10.1007/s10654-023-01047-8 PMC10570208

[6] Mittinty MN, Lynch J. Reflection on modern methods: risk ratio regression—simple concept yet complex computation. Int J Epidemiol. 2023;52(1):309–14. 36416437 10.1093/ije/dyac220 PMC9908057

[7] Deeks JJ. Issues in the selection of a summary statistic for meta-analysis of clinical trials with binary outcomes. Stat Med. 2002;21(11):1575–600. 12111921 10.1002/sim.1188

[8] Gallis JA, Turner EL. Relative measures of association for binary outcomes: challenges and recommendations for the Global Health Researcher. Ann Glob Health. 2019;85(1):137. 31807416 10.5334/aogh.2581 PMC6873895

[9] Turner EL, Platt AC, Gallis JA, Tetreault K, Easter C, McKenzie JE, et al. Completeness of reporting and risks of overstating impact in cluster randomised trials: a systematic review. Lancet Glob Health. 2021;9(8):e1163–8. 34297963 10.1016/S2214-109X(21)00200-X PMC9994534

[10] Daniel R, Zhang J, Farewell D. Making apples from oranges: Comparing noncollapsible effect estimators and their standard errors after adjustment for different covariate sets. Biom J. 2021;63(3):528–57. 33314251 10.1002/bimj.201900297 PMC7986756

[11] Coart E, Bamps P, Quinaux E, Sturbois G, Saad ED, Burzykowski T, Buyse M. Minimization in randomized clinical trials. Stat Med. 2023;42(28):5285–311. 37867447 10.1002/sim.9916

[12] Kahan BC, Morris TP. Adjusting for multiple prognostic factors in the analysis of randomised trials. BMC Med Res Methodol. 2013;13(1):99. 23898993 10.1186/1471-2288-13-99 PMC3733981

[13] Carter K, Scheffold AL, Renteria J, Berger VW, Luo YA, Chipman JJ, Sverdlov O. Regulatory guidance on randomization and the use of randomization tests in clinical trials: a systematic review. Statistics in Biopharmaceutical Research. 2023:1–13.

[14] EMA. DRAFT Qualification opinion for Prognostic Covariate Adjustment (PROCOVA™). In: Health EMASM, editor. Committee for Medicinal Products for Human Use (CHMP). Europe: European Medicines Agency Science Medicines Health; 2022.

[15] Kahan BC, Jairath V, Doré CJ, Morris TP. The risks and rewards of covariate adjustment in randomized trials: an assessment of 12 outcomes from 8 studies. Trials. 2014;15(1):139. 24755011 10.1186/1745-6215-15-139 PMC4022337

[16] Thompson DD, Lingsma HF, Whiteley WN, Murray GD, Steyerberg EW. Covariate adjustment had similar benefits in small and large randomized controlled trials. J Clin Epidemiol. 2015;68(9):1068–75. 25497979 10.1016/j.jclinepi.2014.11.001 PMC5708297

[17] Ge M, Durham LK, Meyer RD, Xie W, Thomas N. Covariate-adjusted difference in proportions from clinical trials using logistic regression and weighted risk differences. Drug Inf J. 2011;45(4):481–93.

[18] Colantuoni E, Rosenblum M. Leveraging prognostic baseline variables to gain precision in randomized trials. Stat Med. 2015;34(18):2602–17. 25872751 10.1002/sim.6507 PMC5018399

[19] Winston L. Agnostic notes on regression adjustments to experimental data: Reexamining Freedman’s critique. Ann Appl Stat. 2013;7(1):295–318.

[20] Group IEEW. ICH Harmonised Tripartite Guideline. Statistical principles for clinical trials. International Conference on Harmonisation E9 Expert Working Group. Stat Med. 1999;18(15):1905–42. 10532877

[21] CPMP. Committee for Proprietary Medicinal Products (CPMP): points to consider on adjustment for baseline covariates. Stat Med. 2004;23(5):701–9. 14981670 10.1002/sim.1647

[22] Raab GM, Day S, Sales J. How to select covariates to include in the analysis of a clinical trial. Control Clin Trials. 2000;21(4):330–42. 10913808 10.1016/s0197-2456(00)00061-1

[23] Senn S. Empirical studies of balance do not justify a requirement for 1,000 patients per trial. . Clin Epidemiol. 2022;22(1878–5921 (Electronic)):S0895–4356. 10.1016/j.jclinepi.2022.02.010 35248697

[24] Janani L, Mansournia MA, Nourijeylani K, Mahmoodi M, Mohammad K. Statistical issues in estimation of adjusted risk ratio in prospective studies. Arch Iran Med. 2015;18(10):713–9. 26443254

[25] Williamson T, Eliasziw M, Fick GH. Log-binomial models: exploring failed convergence. Emerg Themes Epidemiol. 2013;10(1):14. 24330636 10.1186/1742-7622-10-14 PMC3909339

[26] Zou G. A modified poisson regression approach to prospective studies with binary data. Am J Epidemiol. 2004;159(7):702–6. 15033648 10.1093/aje/kwh090

[27] Localio AR, Margolis DJ, Berlin JA. Relative risks and confidence intervals were easily computed indirectly from multivariable logistic regression. J Clin Epidemiol. 2007;60(9):874–82. 17689803 10.1016/j.jclinepi.2006.12.001

[28] Chen W, Shi J, Qian L, Azen SP. Comparison of robustness to outliers between robust poisson models and log-binomial models when estimating relative risks for common binary outcomes: a simulation study. BMC Med Res Methodol. 2014;14:82. 24965498 10.1186/1471-2288-14-82 PMC4079617

[29] Morris TP, Walker AS, Williamson EJ, White IR. Planning a method for covariate adjustment in individually randomised trials: a practical guide. Trials. 2022;23(1):328. 35436970 10.1186/s13063-022-06097-z PMC9014627

[30] Tackney MS MT, White I, Leyrat C, Diaz-Ordaz K, Williamson E. A comparison of covariate adjustment approaches under model misspecification in individually randomized trials. Research Square. 2022. 10.1186/s13063-022-06967-6 PMC9817411 36609282

[31] Norton EC, Miller MM, Kleinman LC. Computing adjusted risk ratios and risk differences in Stata. Stata J. 2013;13(3):492–509.

[32] Tomas J. Aragon MPF, Daniel Wollschlaeger, Adam Omidpanah Tools for training and practicing epidemiologists including methods for two-way and multi-way contingency tables.. In: Omidpanah A, editor. Epidemiology Tools. 0.5–10.1 ed. CRAN2020.

[33] Austin PC, Manca A, Zwarenstein M, Juurlink DN, Stanbrook MB. A substantial and confusing variation exists in handling of baseline covariates in randomized controlled trials: a review of trials published in leading medical journals. J Clin Epidemiol. 2010;63(2):142–53. 19716262 10.1016/j.jclinepi.2009.06.002

[34] Ciolino JD, Palac HL, Yang A, Vaca M, Belli HM. Ideal vs. real: a systematic review on handling covariates in randomized controlled trials. BMC Med Res Methodol. 2019;19(1):136. 31269898 10.1186/s12874-019-0787-8 PMC6610785

[35] Pirondini L, Gregson J, Owen R, Collier T, Pocock S. Covariate adjustment in cardiovascular randomized controlled trials: its value, current practice, and need for improvement. JACC Heart Fail. 2022;10(2213-1787 (Electronic)):297–305. 35483791 10.1016/j.jchf.2022.02.007

[36] Rombach I, Knight R, Peckham N, Stokes JR, Cook JA. Current practice in analysing and reporting binary outcome data—a review of randomised controlled trial reports. BMC Med. 2020;18(1):147. 32507111 10.1186/s12916-020-01598-7 PMC7278160

[37] Wilkinson J, Showell M, Taxiarchi VP, Lensen S. Are we leaving money on the table in infertility RCTs? Trialists should statistically adjust for prespecified, prognostic covariates to increase power. Hum Reprod. 2022;37(5):895–901. 35199145 10.1093/humrep/deac030 PMC9071217

[38] Yu LM, Chan AW, Hopewell S, Deeks JJ, Altman DG. Reporting on covariate adjustment in randomised controlled trials before and after revision of the 2001 CONSORT statement: a literature review. Trials. 2010;11(1):59. 20482769 10.1186/1745-6215-11-59 PMC2886040

[39] Kahan BC, Morris TP. Improper analysis of trials randomised using stratified blocks or minimisation. Stat Med. 2012;31(4):328–40. 22139891 10.1002/sim.4431

[40] Moher D, Liberati A, Tetzlaff J, Altman DG. Preferred reporting items for systematic reviews and meta-analyses: the PRISMA statement. Ann Intern Med. 2009;151(4):264–9, w64. 19622511 10.7326/0003-4819-151-4-200908180-00135

[41] Lefebvre C GJ, Briscoe S, Featherstone R, Littlewood A, Marshall C, Metzendorf M-I, Noel-Storr A, Paynter R, Rader T, Thomas J, Wieland LS. Chapter 4: Searching for and selecting studies. 2022. In: Cochrane Handbook for Systematic Reviews of Interventions version 63 (updated February 2022). Cochrane. Available from: www.training.cochrane.org/handbook.

[42] EndNote. EndNote. EndNote X9 ed. Philadelphia, PA: Clarivate; 2023. p. Thomson Reuters, San Francisco, CA, USA.

[43] Covidence. Covidence systematic review software. Veritas Health Innovation: Melbourne, Australia; 2023.

[44] Matthias Gamer JL, Ian Fellows Puspendra Singh. Coefficients of Interrater Reliability and Agreement for quantitative, ordinal and nominal data: ICC, Finn-Coefficient, Robinson's A, Kendall's W, Cohen's Kappa. CRAN; 2019.

[45] Sjoberg DDWK, Curry M, Lavery JA, Larmarange J. Reproducible summary tables with the gtsummary package. The R Journal. 2021;13(1):570–80.

[46] Team R. RStudio: Integrated Development for R. RStudio, PBC, Boston, MA URL http://www.rstudio.com/. Boston, MA: RStudio PBC; 2024.

[47] Kahan BC, Morris TP. Reporting and analysis of trials using stratified randomisation in leading medical journals: review and reanalysis. BMJ. 2012;345(sep14 1):e5840-e. 22983531 10.1136/bmj.e5840 PMC3444136

[48] Nevins P, Vanderhout S, Carroll K, Nicholls SG, Semchishen SN, Brehaut JC, et al. Review of pragmatic trials found that multiple primary outcomes are common but so too are discrepancies between protocols and final reports. J Clin Epidemiol. 2022;143:149–58. 34896234 10.1016/j.jclinepi.2021.12.006 PMC9058220

[49] Yelland LN, Salter AB, Ryan P. Performance of the modified Poisson regression approach for estimating relative risks from clustered prospective data. Am J Epidemiol. 2011;174(8):984–92. 21841157 10.1093/aje/kwr183

[50] Huang FL. Alternatives to logistic regression models in experimental studies. J Exp Educ. 2022;90(1):213–28.

