# Supplementary material for: Estimating relative risks and risk differences in randomised controlled trials: a systematic review of current practice
Source: Trials. 2025 Jan 2;26:1. doi: 10.1186/s13063-024-08690-w (PMC11694472; doi:10.1186/s13063-024-08690-w)
Supplement: Supplementary file 1 — Supplementary Material 1: Table A2: Summary of the search strategy and papers identified via Ovid Medline. Table A3: Data Dictionary Codebook for review of binary outcomes. Table A4: Discrepancies and consensus agreement. Table A5: Detailed description of methods for estimating relative risks from overall and nested sample. Table A6: Detailed description of methods for estimating risk differences from overall and nested sample. [file 13063_2024_8690_MOESM1_ESM.docx]

# Appendix 1

Table A1: Protocol for a systematic review of current practice of estimating relative risks and risk differences in randomised controlled trials

| **Introduction**  CONSORT guidance recommends the reporting of relative risks and risk differences as complementary measures of effect for binary outcomes (Moher et al., 2010). In tandem, the European Medicines Agency (EMA) and US Food and Drug Administration (FDA) also recommend adjusting for pre-specified covariates when estimating treatment effects in randomised trials (Agency, 2015) to improve statistical efficiency (Office of the Federal Register, 2021, Benkeser et al., 2020, Steingrimsson et al., 2017). The unadjusted and adjusted odds ratio (aOR) are commonly reported in medical research (Ackerman et al., 2021). Whilst the odds ratio (OR) can be readily estimated using logistic regression, it is not an intuitive summary measure (Deeks, 2002), and the magnitude of the intervention effect can be overstated if the odds ratios are misunderstood (Turner et al., 2021). Furthermore, the OR is non-collapsible, meaning that adjusting for covariates changes the meaning of the estimate (the unadjusted OR targets the marginal estimand, while the adjusted OR represents the conditional estimand) (Daniel et al., 2021). In comparison, relative risks (RR) and risk differences (RD) are easier to interpret, meaning they can facilitate the translation of findings into practice and reduce misinterpreting the magnitude of the treatment effect (Moher et al., 2010). However, the practice of adjusting for covariates when estimating RR and RDs are fraught with challenges (Senn, 2022, Janani et al., 2015). So, although there is a growing appreciation for reporting adjusted RR and RD (Pirondini et al., 2022, Wilkinson et al., 2022, Rombach et al., 2020, Ciolino et al., 2019, Austin et al., 2010), some issues persist as barriers. For example, when fitting binomial regression with log or identity links to estimate the adjusted relative risk (aRR) or risk difference (aRD), models do not always converge (Williamson et al., 2013, Gallis and Turner, 2019). While other less-known approaches are available, they are not without their limitations. For example, modified Poisson can yield probabilities that are greater than 1 (Localio et al., 2007, Chen et al., 2014), generalised estimation equations are prone to non-convergence (Gallis and Turner, 2019), and bootstrapping is computationally intensive (Fang, 2011, Petersen and Deddens, 2008). Furthermore, it is worth noting that reporting adjusted relative risks and risk differences is not commonplace, and the methods available might be under-appreciated (Pirondini et al., 2022, Wilkinson et al., 2022, Rombach et al., 2020, Ciolino et al., 2019, Austin et al., 2010). Findings from previous reviews of practice of about a decade (from 2007 and 2019) (Pirondini et al., 2022, Wilkinson et al., 2022, Rombach et al., 2020, Ciolino et al., 2019, Austin et al., 2010) do not show major changes in approaches used to estimate binary outcomes. See Table 1 for details. What is clear is that many of the RCTs included in these reviews are two-arm superiority trials that frequently evaluate binary outcomes (with frequency ranging from 28.1% to 72%). However, covariate adjustment was performed in about a third of trials, possibly slightly less for binary outcomes and even less for relative risks or risk differences. Adjustment was also more common when covariates were included in the allocation procedure or to account for baseline values of covariates. There is insufficient detail on methods for estimating covariate-adjusted RR or RD to understand how covariate adjustment is implemented comprehensively, its impact on the point estimate, and improvement in precision, efficiency and power. Thus, it is unclear how often these alternative approaches are applied in practice or if other barriers exist, such as a lack of awareness or capacity development.  The aim is to evaluate the current practice of estimating covariate-adjusted RR or RDs and determine the practical impact of covariate adjustment. Using individually randomised controlled trials (RCTs) with a binary primary outcome, published in a selection of high-impact journals, that report a RR or RDs for the primary outcome, the objectives are to:  **Stage I: Overall review sample**   - Objective 1: Estimate the proportion that report a covariate-adjusted RR or RD within a primary, secondary or exploratory analysis.   **Stage II: Nested sample; a sub-sample of RCTs identified from the overall review sample that adjusts for covariates:**   - Objective 2: Identify methods used to estimate the covariate-adjusted RR and RD and the proportion that use such methods. - Objective 3: Describe how covariates are chosen for adjustment, including pre-specification of clinically important variables or data-driven approaches. - Objective 4: Determine the realised impact of covariate adjustment by comparing unadjusted and adjusted point estimates, confidence intervals (CIs) or standard errors (SEs) and p-values.   **Methods for the systematic review**  **Design**  Since this review focuses on RCT methodology, it is not eligible for registration on PROSPERO, but nonetheless, the protocol was developed using Preferred Reporting Items for Systematic Reviews Meta-Analyses (PRISMA) guidelines (Moher D, 2009). Cochrane recommendations (Lefebvre C, 2022) were incorporated during the design of the search strategy, and findings from previous reviews (Rombach et al., 2020, Ciolino et al., 2019) informed the type of information extracted. Searches were limited to individual RCTs published in four high-ranking journals that publish across diverse fields. The journals include the New England Journal of Medicine (NEJM), the Journal of the American Medical Association (JAMA), the Lancet, and the British Medical Journal (BMJ). These journals were chosen because adherence to CONSORT recommendations should be higher within these journals, and therefore, reporting of RR or RD as a summary measure for a binary primary outcome should be more frequent than in lower-impact journals.  **Search strategy**  A combination of keywords and Boolean operators were used to search one primary bibliographic database -Ovid Medline. Therefore, to maximise the sensitivity and precision of the search strategy (Lefebvre C, 2022), search strings were developed using recommended filters (Lefebvre C, 2022) to identify RCTs published between 2018 and 2023. This timeframe was considered sufficient to identify the required number of studies to evaluate the impact of covariate adjustment as described under the justification for sample size requirements. See Appendix 1 for details of the search strategy. All RCTs identified via the search strings within the specified timeframe were screened for inclusion against the phased eligibility criteria for the first and second part of the review. The authors will independently perform the data extraction in duplicate.  **Eligibility criteria**  All two-arm, individually randomised controlled trials conducted among humans in selected high-ranking journals namely, the NEJM, JAMA, the Lancet and the BMJ - that met the following criteria were included:  **Inclusion criteria**  **Overall review sample**   1. RCTs published between January 1, 2018, and March 11, 2023, that report a binary primary outcome and RR or RD as a summary measure.   **Nested review sample**   1. The sub-sample of RCTs identified from the overall review sample that report a covariate-adjusted RR or RD for a primary, secondary or exploratory analysis.   **Exclusion criteria**  **The criteria for excluding studies or RCTs from the overall review sample are as follows:**   1. All non-human, vaccine, or drug safety trials. 2. Cluster or cross-over randomised studies, pilot, feasibility, phase I or II trials, and trials with multiple arms (>2 arms) or factorial trials. 3. RCTs identified that are not primary reports but secondary publications (i.e., follow-up reports), publications primarily reporting health economic analyses or bolt-on studies conducted alongside RCTs. 4. RCTs identified that only report proportions (i.e., no treatment effect), odds ratios, or other measures of effect that are not relative risks or risk differences. 5. RCTs identified that evaluate co-primary outcomes (e.g., two or more distinct outcome variables) or multiple primary outcomes. 6. RCTs identified that evaluate other primary outcomes, i.e., continuous, rate, ordinal, count, or time-to-event outcomes. 7. Duplicate publications, abstracts, ongoing studies, conference proceedings, research letters, commentaries, editorials, or review articles.   **Criteria for excluding studies or RCTs within the nested sample**   1. RCTs in the nested sample that do not report any values of either the point estimate, CI and p-value for covariate-adjusted RR or RD within the main report for the primary, secondary or exploratory analysis. RCTs that provide at least one of these values will be included.   **Sample size justification**  We aimed to identify about 300 studies that meet our inclusion criteria (two-arm RCTs with a binary outcome reporting an RR or RD as a summary measure). To identify about 300 studies that meet these broad inclusion criteria, we anticipate we need to screen about 900 RCT abstracts (since about one-third of RCTs report a binary primary outcome and an RR or RD (Rombach et al., 2020)). We estimate that approximately 245 RCTs are published each year in the four target journals, the equivalent of 980 RCTs. Thus, to achieve a target of ~900 RCTs, we estimate we need to search from 2018 to 2023. Assuming approximately 30% of RCTs implement covariate adjustment (Ciolino et al., 2019), a sample size of 300 would allow us to estimate the percentage of studies implementing covariate adjustment (~30%) with a 95% CI of 25% to 35% (objective 1). Furthermore, identifying 100 studies from the initial sample of 300 that implement covariate adjustment will be of reasonable size to allow us to summarise how covariate-adjusted RR or RD is estimated and pre-specification of covariates for adjustment is implemented across identified RCTs (to evaluate objectives 2 and 3) and qualitatively compare the unadjusted and covariate-adjusted results (objective 4).  **Screening and selection**  The PRISMA flow diagram illustrates stages of eligibility assessments for RCTs included in the review with reasons for exclusion. Records identified will be imported into a bibliographic referencing software programme (EndNote, 2023). One reviewer (JT) will independently screen the titles and abstracts for relevance using Covidence systematic review software (Covidence, 2023). The total number of eligible RCTs identified at the initial stage will form the ‘overall review sample’.  **Overall review sample**   1. The initial process will involve screening the title and abstracts of approximately 900 RCTs to identify whether an RR or RD is reported as a summary measure for the primary outcome. Our working definition of the primary outcome uses the hierarchy: that is described by the study authors as the primary outcome, or in the absence of a clear specification of the primary outcome, we use that used for sample size calculation, or the first outcome reported in the abstract. For those RCTs which are identified as meeting the eligibility criteria and having a binary primary outcome, all full texts will be retrieved to identify if an RR or RD was reported. A study with an unknown trial phase will be included if it meets all other criteria for this phase. 2. For all trials that meet the eligibility criteria for inclusion in the overall review sample (~300 trials), we will determine if covariate adjustment was implemented within the primary, secondary or exploratory analysis.   **Nested study sample**   1. All trials identified from the overall sample that implements covariate-adjustment will be considered for this stage of the review. A more detailed extraction will then be undertaken using the full texts of all trials that meet all eligibility criteria for the nested sample (that is, two-arm RCTs with a binary primary outcome that report a covariate-adjusted RR or RD), which should be approximately 100.   **Data extraction**  **Overall review sample**  At this stage of the review, we will extract basic trial information the following information will be extracted from each study eligible for inclusion:   1. Information about the authors, year of publication, the title of the journal 2. Information on study characteristics related to study design and the number of study centres. 3. Identifying whether a primary binary outcome was reported and, if so, what type of summary measure, i.e., RR or RD. 4. Whether a covariate-adjusted RR, RD or both, is reported for the primary, secondary or exploratory analysis. We will also extract information on whether covariate-adjusted ORs are reported.   **Nested review sample**  For the nested sample, a more detailed extraction will be undertaken to retrieve information such as the type and number of covariates used during randomisation and covariate adjustment analysis. The additional information will include:   1. When reported, the method used to estimate the unadjusted and adjusted RR or RD. 2. The total sample size and sample size per arm (randomised and analysed) for the primary unadjusted and covariate-adjusted analyses. 3. The number or proportion of events per arm in the primary unadjusted and covariate-adjusted analyses. 4. The covariates used during randomisation and type of randomisation scheme (i.e., minimisation, stratification or other methods). 5. The type and number of additional pre-specified covariates used for adjustment. 6. How covariates were chosen, including selection based on the pre-specification of clinically important variables, data-driven approaches (accounting for variables used during randomisation) or if this was unclear. 7. The values of the point estimate and measures of uncertainty such as CI, SE and p-value for the unadjusted and adjusted RR or RD. Data will be extracted for all covariate-adjusted estimates reported. Additional details, such as non-convergence or unobserved events, will be extracted from studies that provide this information.   Data will be extracted using a standardised data extraction proforma built-in Airtable. The proforma will undergo iterative review by the study team to address discrepancies and ensure consistency. One review author will extract data from studies, and another author will independently check these data for accuracy.  **Data synthesis**  After data extraction is complete, findings will be described using tables and figures — proportions (percentages) for categorical data or medians (interquartile ranges) for continuous data. All descriptive, exploratory analysis and data visualisations will be performed using R version x64 3.5.1 (R Foundation for Statistical Computing, Vienna, Austria). |
| --- |

Table A2: Summary of the search strategy and papers identified via Ovid Medline

|  | Ovid MEDLINE(R) and In-Process, In-Data-Review & Other Non-Indexed Citations <1946 to March 31, 2023> | |
| --- | --- | --- |
| 1 | randomized controlled trial.pt. or random*.ti. or (random* and (trial or placebo)).ti,ab. | 883120 |
| 2 | ("New England Journal of Medicine" or "JAMA" or "BMJ" or "Lancet").jt. | 399798 |
| 3 | 1 and 2 | 15806 |
| 4 | (systematic review or meta-analysis or review or comment or letter or editorial or news).pt. or (review or meta-analysis or metaanalysis).ti. | 6086354 |
| 5 | exp animals/ not humans.sh. | 5209414 |
| 6 | 4 or 5 | 11032572 |
| 7 | (2018* or 2019* or 2020* or 2021* or 2022* or 2023 Mar*).dp,ep. | 7211030 |
| 8 | 3 not 6 | 13104 |
| 9 | 7 and 8 | 1920 |

**Key:** ab – abstract, dp – date of publication, ep –electronic date of publication, jt – journal title, pt – publication type, sh – subject heading, ti – title, *- truncation

Table A3: Data Dictionary Codebook for review of binary outcomes

| **#** | **Field Label** | | **Field Attributes**  **(Field Type, Validation, Choices)** | **Skip Logic** |
| --- | --- | --- | --- | --- |
| **Screening questions** | | | | |
|  | Covidence ID | | Unique number identifier |  |
|  | reviewer_name | | Name of the person reviewing the article. | - Go to 1 |
|  | Is the study a randomised controlled trial (RCT)? | | 1 Yes  0 No | - Go to 2 - Exclude the study |
|  | Is the study a cluster or cross-over trial? | | 1 Yes  0 No | - Exclude the study - Go to 3 |
|  | Is the study a phase I, phase II, vaccine (human and non-human), drug safety, pilot or feasibility trial? | | 1 Yes  0 No | - Exclude the study - Go to 4 |
|  | Is the study a trial with a single arm or multiple arms (>2 arms) or a factorial trial? | | 1 Yes  0 No | - Exclude the study - Go to 5 |
|  | Is the trial a bolt-on study, an economic evaluation, secondary data, post-hoc or follow-up analysis? | | 1 Yes  0 No | - Exclude the study - Go to 6 |
|  | Is the trial an abstract, an ongoing study, a conference paper/proceeding, a research letter, a commentary or an editorial? | | 1 Yes  0 No | - Exclude the study - Go to 7 |
|  | What outcome have you identified as the primary outcome of the trial? | | Text (notes): | - Go to 8 |
|  | Select an option that indicates how the outcome was identified as the primary outcome. | | 1 Outcome described as primary by study authors  2 Outcome used in sample size calculation  3 First outcome listed in the abstract | - Go to 9 |
|  | Does the trial evaluate a continuous, rate, ordinal, count, or time-to-event primary outcome? | | 1 Yes  0 No | - Exclude the study - Go to 10 |
|  | Does the trial evaluate a primary binary outcome? | | 1 Yes  0 No | - Go to 11 - Exclude the study |
|  | Does the trial evaluate a co-primary binary outcome? | | 1 Yes  0 No | - Exclude the study - Go to 12 |
|  | Does the trial report the primary outcome with no treatment effect (i.e., outcomes reported as proportions alone)? | | 1 Yes  0 No | - Exclude the study - Go to 13 |
|  | Was a relative risk (RR) reported as a summary measure(s) for the primary outcome? | | 1 Yes  0 No | - Study eligible for inclusion and data extraction. Go to 14. - Exclude the study |
|  | Was a risk difference (RD) reported as a summary measure(s) for the primary outcome? | | 1 Yes  0 No | - Study eligible for inclusion and data extraction. Go to 15. - Exclude the study |
|  | Was an odd ratio (OR) reported as a summary measure(s) for the primary outcome, in addition to an RR or RD? | | 1 Yes  0 No | - Study eligible for inclusion. Go to data extraction. - Exclude the study |
| ***Sections for data extraction*** | | | | |
| **#** | **Variable /**  **Field Name** | **Field Label** | **Field Attributes**  **(Field Type, Validation, Choices)** | - **Skip Logic** |
| **The next set of questions focuses on data extraction for the characteristics of the overall review sample.** | | | | |
|  | ***Study characteristics and randomisation process*** | | | |
|  | no_centres_rprt | Does the study report the number of centres included? | 1 Yes  0 No | - Go to 2 - Go to 3 |
|  | type_centre | Is the study a multi-centre trial? | 1 Yes  0 No | - Go to 3 - Go to 3 |
|  | SS_arm_rprt | Does the study report the number of participants randomised to each arm? | 1 Yes  0 No | - Go to 4 - Go to 6 |
|  | SS_int_arm | What is the number of participants randomised to the *intervention* arm? | Number (integer): | - Go to 5 |
|  | SS_cont_arm | What is the number of participants randomised to the *control* arm? | Number (integer): | - Go to 6 |
| **Subsection for detailed data extraction from the nested sample** | | | | |
| The objective is to elicit what the authors did for the primary analysis. If the primary analysis is unclear, please extract data from the analysis identified as the main result or first analysis. If both ITT and per-protocol analyses are reported for the primary analysis, extract data for the unadjusted and all covariate-adjusted analyses under ITT analysis. | | | | |
| **Relative risk (RR)** | | | | |
|  | ***The next set of questions is about how the authors estimate the unadjusted relative risk (RR).*** | | | |
|  | RR_rprt | Was an unadjusted RR reported for the primary outcome? | 1 Yes  0 No | - Go to 7 - Go to 23 |
|  | RR_methd | What method was used to estimate the unadjusted RR? | Select all that apply:  1 Log-binomial model  2 Crude estimator  3 Ratio of proportions + Chi-squared  888 Unclear / Not reported / Unable to determine / Missing  999 Other (specify) | - Go to 9 - Go to 8 |
|  | RR_methd_other | If ‘Other’ was selected above, specify the other method used to estimate the unadjusted RR. | Text (notes): | - Go to 9 |
|  | ***The next set of questions is about the estimates reported for the unadjusted RR.*** | | | |
|  | RR_unadj_est_rprt | Does the study report the point estimate for the unadjusted RR? | 1 Yes  0 No | - Go to 10 - Go to 11 |
|  | RR_unadj_est | What is the point estimate for the unadjusted RR? | Number (decimal): | - Go to 11 |
|  | RR_unadj_se_rprt | Does the study report the standard error (SE) for the unadjusted RR? | 1 Yes  0 No | - Go to 12 - Go to 13 |
|  | RR_unadj_se | What is the SE for the unadjusted RR? | Number (decimal): | - Go to 21 |
|  | RR_unadj_lci_rprt | Does the study report a lower confidence interval for the unadjusted RR? | 1 Yes  0 No | - Go to 14 - Go to 15 |
|  | RR_unadj_lci | What is the lower confidence interval for the unadjusted RR? | Number (decimal): | - Go to 17 |
|  | RR_unadj_lci_inf_rprt | Does the study report infinity as the lower confidence interval for the unadjusted RR? | 1 Yes  0 No | - Go to 16 - Go to 17 |
|  | RR_unadj_lci_inf | What is the lower confidence interval for the unadjusted RR? | Number (decimal): | - Go to 17 |
|  | RR_unadj_uci_rprt | Does the study report the upper confidence interval for the unadjusted RR? | 1 Yes  0 No | - Go to 18 - Go to 19 |
|  | RR_unadj_uci | What is the upper confidence interval for the unadjusted RR? | Number (decimal): | - Go to 21 |
|  | RR_unadj_uci_inf_rprt | Does the study report infinity as the upper confidence interval for the unadjusted RR? | 1 Yes  0 No | - Go to 20 - Go to 21 |
|  | RR_unadj_uci_inf | What is the upper confidence interval for the unadjusted RR? | Number (decimal): | - Go to 21 |
|  | RR_unadj_pval_rprt | Does the study report the p-value for the unadjusted RR? | 1 Yes  0 No | - Go to 22 - Go to 23 |
|  | RR_unadj_pval | What is the p-value for the unadjusted RR? | Number (decimal): | - Go to 23 |
|  | ***The next set of questions is about how the authors estimate the adjusted RR.*** | | | |
|  | aRR_anlys_rprt | Was an adjusted RR reported for the primary outcome? | 1 Yes  0 No | - Go to 24 - Go to 35 |
|  | rand_covrr | Were covariates used in the randomisation process, e.g., stratification, minimisation? | 1 Yes  0 No  888 Unclear / Not reported / Unable to determine / Missing | - Go to 25 - Go to 27 - Go to 27 |
|  | no_rand_covrr | What is the total number of covariate(s) used in the randomisation process? | Integer (Min: 0, Max: 100): | - Go to 26 |
|  | rand_cov_adjrr | Were all covariates used in the randomisation process included in the adjusted analysis? | 1 Yes (complete)  2 Partial  0 None  888 Unclear / Not reported / Unable to determine / Missing | - Go to 27 |
|  | cov_adjrr | Were covariates (besides those used during randomisation, if any) included in the adjusted RR analysis? | 1 Yes  0 No  888 Unclear / Not reported / Unable to determine / Missing | - Go to 28 - Go to 32 - Go to 32 |
|  | tot_no_cov_arr_rprt | Does the study report the total number of covariate(s) included in the adjusted RR analysis? | 1 Yes  0 No  888 Unclear / Not reported / Unable to determine / Missing | - Go to 29 - Go to 30 - Go to 30 |
|  | tot_no_cov_arr | What is the total number of covariate(s) used in the adjusted RR analysis? | Integer (Min: 0, Max: 100): | - Go to 30 |
|  | reason_aRR | What was the reason(s) for including covariates in the adjusted RR analysis? | Select all that apply:  1 Pre­specification (variables that were used during the randomisation or chosen using historical evidence)  2 Data-driven ­ post-hoc (e.g., lack of balance / statistical significance)  888 Unclear / Not reported / Unable to determine / Missing  999 Other (specify) | - Go to 32 - Go to 32 - Go to 32 - Go to 31 |
|  | other_reason_aRR | If ‘Other’ was selected above, what was the other reason(s) (besides those listed above) for including covariates in the adjusted RR analysis? | Text (notes): e.g., other variable selection approaches | - Go to 32 |
|  | aRR_anlys | Was the adjusted RR reported for the primary, secondary or exploratory analysis? | Select all that apply:  1 Primary analysis  2 Secondary analysis  3 Exploratory analysis  888 Unclear / Not reported / Unable to determine / Missing | - Go to 33 |
|  | aRR_methd | What method was used to estimate the adjusted RR? | Select all methods that apply:  1 Log-binomial model  2 Modified Poisson model + Robust/sandwich estimator  3 Marginal standardisation + Delta-method  4 Log-binomial model + Permutation test  5 Ratio of proportions + Chi-squared  888 Unclear / Not reported / Unable to determine / Missing  999 Other (specify) | - Go to 35 - Go to 34 |
|  | aRR_methd_other | If ‘Other’ was selected above, specify the other method used to estimate the adjusted RR. | Text (notes): | - Go to 35 |
|  | ***The next set of questions is about the estimates reported for the adjusted RR.*** | | | |
|  | RR_adj_est_rprt | Does the study report the point estimate for the adjusted RR? | 1 Yes  0 No | - Go to 36 - Go to 37 |
|  | RR_adj_est | What is the point estimate for the adjusted RR? | Number (decimal): | - Go to 37 |
|  | RR_adj_se_rprt | Does the study report the SE for the adjusted RR? | 1 Yes  0 No | - Go to 38 - Go to 39 |
|  | RR_adj_se | What is the SE for the adjusted RR? | Number (decimal): | - Go to 47 |
|  | RR_adj_lci_rprt | Does the study report a lower confidence interval for the adjusted RR? | 1 Yes  0 No | - Go to 40 - Go to 41 |
|  | RR_adj_lci | What is the lower confidence interval for the adjusted RR? | Number (decimal): | - Go to 41 |
|  | RR_adj_lci_inf_rprt | Does the study report infinity as the lower confidence interval for the adjusted RR? | 1 Yes  0 No | - Go to 42 - Go to 43 |
|  | RR_adj_lci_inf | What is the lower confidence interval for the adjusted RR? | Number (decimal): | - Go to 43 |
|  | RR_adj_uci_rprt | Does the study report the upper confidence interval for the adjusted RR? | 1 Yes  0 No | - Go to 44 - Go to 45 |
|  | RR_adj_uci | What is the upper confidence interval for the adjusted RR? | Number (decimal): | - Go to 47 |
|  | RR_adj_uci_inf_rprt | Does the study report infinity as the upper confidence interval for the adjusted RR? | 1 Yes  0 No | - Go to 46 - Go to 47 |
|  | RR_adj_uci_inf | What is the upper confidence interval for the adjusted RR? | Number (decimal): | - Go to 47 |
|  | RR_adj_pval_rprt | Does the study report the p-value for the adjusted RR? | 1 Yes  0 No | - Go to 48 - Go to 49 |
|  | RR_adj_pval | What is the p-value for the adjusted RR? | Number (decimal): | - Go to 49 |
| **Risk difference (RD)** | | | | |
|  | ***The next set of questions is about how the authors estimate the unadjusted risk difference (RD)*** | | | |
|  | RD_rprt | Was an unadjusted RD reported for the primary outcome? | 1 Yes  0 No | - Go to 50 - Go to 51 |
|  | RD_methd | What method was used to estimate the unadjusted RD? | Select all that apply:  1 Binomial model  2 Crude estimator  3 Difference in proportions + Chi-squared  888 Unclear / Not reported / Unable to determine / Missing  999 Other (specify) | - Go to 52 - Go to 51 |
|  | RD_methd_other | If ‘Other’ was selected above, specify the other method used to estimate the unadjusted RD. | Text (notes): | - Go to 52 |
|  | ***The next set of questions is about the estimates reported for the unadjusted RD.*** | | | |
|  | RD_unadj_est_rprt | Does the study report the point estimate for the unadjusted RD? | 1 Yes  0 No | - Go to 53 - Go to 54 |
|  | RD_unadj_est | What is the point estimate for the unadjusted RD? | Number (decimal): | - Go to 54 |
|  | RD_unadj_se_rprt | Does the study report a SE for the unadjusted RD? | 1 Yes  0 No | - Go to 55 - Go to 56 |
|  | RD_unadj_se | What is the SE for the unadjusted RD? | Number (decimal): | - Go to 64 |
|  | RD_unadj_lci_rprt | Does the study report a lower confidence interval for the unadjusted RD? | 1 Yes  0 No | - Go to 57 - Go to 58 |
|  | RD_unadj_lci | What is the lower confidence interval for the unadjusted RD? | Number (decimal): | - Go to 58 |
|  | RD_unadj_lci_rprt_inf | Does the study report infinity as the lower confidence interval for the unadjusted RD? | 1 Yes  0 No | - Go to 59 - Go to 60 |
|  | RD_unadj_lci_inf | What is the lower confidence interval for the unadjusted RD when the value is infinity? | Text (notes): e.g., infinity | - Go to 60 |
|  | RD_unadj_uci_rprt | Does the study report an upper confidence interval for the unadjusted RD? | 1 Yes  0 No | - Go to 61 - Go to 62 |
|  | RD_unadj_uci | What is the upper confidence interval for the unadjusted RD? | Number (decimal): | - Go to 64 |
|  | RD_unadj_uci_rprt_inf | Does the study report infinity as the upper confidence interval for the unadjusted RD? | 1 Yes  0 No | - Go to 63 - Go to 64 |
|  | RD_unadj_uci_inf | What is the upper confidence interval for the unadjusted RD when the value is infinity? | Text (notes): e.g., infinity | - Go to 64 |
|  | RD_unadj_pval_rprt | Does the study report a p-value for the unadjusted RD? | 1 Yes  0 No | - Go to 65 - Go to 66 |
|  | RD_unadj_pval | What is the p-value for the unadjusted RD? | Number (decimal): | - Go to 66 |
|  | ***The next set of questions is about how the authors estimate the adjusted RD.*** | | | |
|  | aRD_anlys_rprt | Was an adjusted RD reported for the primary outcome? | 1 Yes  0 No | - Go to 67 - Go to 78 |
|  | rand_covrd | Were covariates used in the randomisation process, e.g., stratification, minimisation? | 1 Yes  0 No  888 Unclear / Not reported / Unable to determine / Missing | - Go to 68 - Go to 70 - Go to 70 |
|  | no_rand_covrd | What is the total number of covariate(s) used in the randomisation process? | Integer (Min: 0, Max: 100): | - Go to 69 |
|  | rand_cov_adjrd | Were all covariates used in the randomisation process included in the adjusted analysis? | 1 Yes (complete)  2 Partial  0 None  888 Unclear / Not reported / Unable to determine / Missing | - Go to 70 |
|  | cov_adjrd | Were covariates (besides those used during randomisation, if any) included in the adjusted RD analysis? | 1 Yes  0 No  888 Unclear / Not reported / Unable to determine | - Go to 71 - Go to 73 - Go to 73 |
|  | tot_no_cov_ard_rprt | Does the study report the total number of covariate(s) used in the adjusted RD analysis? | 1 Yes  0 No  888 Unclear / Not reported / Unable to determine / Missing | - Go to 72 - Go to 73 - Go to 73 |
|  | tot_no_cov_ard | What is the number of total covariate(s) used in the adjusted RD analysis? | Integer (Min: 0, Max: 100): | - Go to 73 |
|  | reason_aRD | What was the reason(s) for including variables in the adjusted RD analysis? | Select all that apply:  1 Pre­specification (efficiency)  2 Data-driven ­ post-hoc (e.g., lack of balance/ Statistical significance)  888 Unclear / Not reported / Unable to determine / Missing  999 Other (specify) | - Go to 75 - Go to 75 - Go to 75 - Go to 74 |
|  | other_reason_aRD | If ‘Other’ was selected above, what was the reason(s) (besides those listed above) for including variables in the adjusted RD analysis? | Text (notes): e.g., other variable selection approaches | - Go to 75 |
|  | aRD_anlys | Was the adjusted RD reported for the primary, secondary or exploratory analysis? | Select all that apply:  1 Primary analysis  2 Secondary analysis  3 Exploratory analysis  888 Unclear / Not reported / Unable to determine / Missing | - Go to 76 |
|  | aRD_methd | What method was used to estimate the adjusted RD? | Select all that apply:  1 Binomial model, identity link  2 Modified Poisson identity model + Robust / sandwich estimator  3 Marginal standardisation + Delta-method  4 Linear model + Robust / sandwich estimator  5 Binomial model, identity link + Permutation test  6 Difference in proportions + Chi-squared  888 Unclear / Not reported / Unable to determine / Missing  999 Other (specify) | - Go to 78 - Go to 77 |
|  | aRD_methd_other | If ‘Other’ was selected above, specify the method used to estimate the adjusted RD. | Text (notes): | - Go to 78 |
|  | ***The next set of questions is about the estimates reported for the adjusted RD.*** | | | |
|  | RD_adj_est_rprt | Does the study report the point estimate for adjusted RD? | 1 Yes  0 No | - Go to 79 - Go to 80 |
|  | RD_adj_est | What is the point estimate for the adjusted RD? | Number (decimal): | - Go to 80 |
|  | RD_adj_se_rprt | Does the study report an SE for the adjusted RD? | 1 Yes  0 No | - Go to 81 - Go to 82 |
|  | RD_adj_se | What is the SE for the adjusted RD? | Number (decimal): | - Go to 91 |
|  | RD_adj_lci_rprt | Does the study report a lower confidence interval for the adjusted RD? | 1 Yes  0 No | - Go to 83 - Go to 84 |
|  | RD_adj_lci | What is the lower confidence interval for the adjusted RD? | Number (decimal): | - Go to 86 |
|  | RD_adj_lci_rprt_inf | Does the study report infinity as the lower confidence interval for the adjusted RD? | 1 Yes  0 No | - Go to 85 - Go to 86 |
|  | RD_adj_lci_inf | What is the lower confidence interval for the adjusted RD when the value is infinity? | Text (notes): e.g., infinity | - Go to 86 |
|  | RD_adj_uci_rprt | Does the study report an upper confidence interval for the adjusted RD? | 1 Yes  0 No | - Go to 87 - Go to 88 |
|  | RD_adj_uci | What is the upper confidence interval for the adjusted RD? | Number (decimal): | - Go to 90 |
|  | RD_adj_uci_rprt_inf | Does the study report infinity as the upper confidence interval for the adjusted RD? | 1 Yes  0 No | - Go to 89 - Go to 90 |
|  | RD_adj_uci_inf | What is the upper confidence interval for the adjusted RD when the value is infinity? | Text (notes): e.g., infinity | - Go to 90 |
|  | RD_adj_ pval_rprt | Does the study report a p-value for the adjusted RD? | 1 Yes  0 No | - Go to 91 - Go to 92 |
|  | RD_adj_pval | What is the p-value for the adjusted RD? | Number (decimal): | - Go to 92 |
|  | comments | Add any other comment about eligible studies. | Text (notes): e.g., non-convergence | - Go to 93 |
|  | DE_complete | Data extraction is complete? | 0 Incomplete  1 Complete | End |

Table A4: Discrepancies and consensus agreement

| **No. of discrepancies** | **Covidence #** | **SR Variable** | **JT data extractions** | **LM data extractions** | **Consensus** |
| --- | --- | --- | --- | --- | --- |
|  | #112 | Was a relative risk (RR) reported as a summary measure(s) for the primary outcome? | 1 Yes | 0 No | 0 No |
|  | #210 | Was a relative risk (RR) reported as a summary measure(s) for the primary outcome? | 1 Yes | 0 No | 0 No |
|  | #943 | Was a relative risk (RR) reported as a summary measure(s) for the primary outcome? | 1 Yes | 0 No | 0 No |
|  | #980 | Was a relative risk (RR) reported as a summary measure(s) for the primary outcome? | 1 Yes | 0 No | 0 No |
|  | #112 | Was a risk difference (RD) reported as a summary measure(s) for the primary outcome? | 0 No | 1 Yes | 1 Yes |
|  | #210 | Was a risk difference (RD) reported as a summary measure(s) for the primary outcome? | 0 No | 1 Yes | 1 Yes |
|  | #677 | Was a risk difference (RD) reported as a summary measure(s) for the primary outcome? | 1 Yes | 0 No | 0 No |
|  | #980 | Was a risk difference (RD) reported as a summary measure(s) for the primary outcome? | 0 No | 1 Yes | 1 Yes |
|  | #210 | Was an odd ratio (OR) reported as a summary measure(s) for the primary outcome, in addition to an RR or RD? | 0 No | 1 Yes | 1 Yes |
|  | #423 | Was an odd ratio (OR) reported as a summary measure(s) for the primary outcome, in addition to an RR or RD? | 1 Yes | 0 No | 1 Yes |
|  | #670 | Was an odd ratio (OR) reported as a summary measure(s) for the primary outcome, in addition to an RR or RD? | 1 Yes | 0 No | 0 No |
|  | #918 | Was an odd ratio (OR) reported as a summary measure(s) for the primary outcome, in addition to an RR or RD? | 1 Yes | 0 No | 0 No |
|  | #980 | Was an odd ratio (OR) reported as a summary measure(s) for the primary outcome, in addition to an RR or RD? | 1 Yes | 0 No | 0 No |
|  | #1049 | Was an odd ratio (OR) reported as a summary measure(s) for the primary outcome, in addition to an RR or RD? | 1 Yes | 0 No | 1 Yes |
|  | #1072 | Was an odd ratio (OR) reported as a summary measure(s) for the primary outcome, in addition to an RR or RD? | 1 Yes | 0 No | 1 Yes |
|  | #1106 | type_centre | 1 Yes | 0 No | 1 Yes |
|  | #295 | SS_int_arm | 558 | 381 | 381 |
|  | #925 | SS_int_arm | 677 | 678 | 678 |
|  | #1106 | SS_int_arm | 91 | 89 | 91 |
|  | #295 | SS_cont_arm | 548 | 376 | 376 |
|  | #925 | SS_cont_arm | 678 | 677 | 677 |
|  | #677 | RR_rprt | 1 Yes | 0 No | 1 Yes |
|  | #423 | RR_methd | 4 Ratio of proportions + Chi-squared test | 888 Unclear / Not reported / Unable to determine / Missing | We assume that a crude estimator was used to derive the point estimate, the approach for the 95% CI is unclear, and the χ2 / Fishers exact was used for the p-value |
|  | #474 | RR_methd | 999 Other (specify) | 2 Crude estimator ratio of proportion | We assume that a crude estimator was used to derive the point estimate, the approach for the 95% CI is unclear, and the χ2 / Fishers exact was used for the p-value. |
|  | #925 | RR_methd | 4 Ratio of proportions + Chi-squared test | 888 Unclear / Not reported / Unable to determine / Missing | We assume that a crude estimator was used to derive the point estimate, the approach for the 95% CI is unclear, and the χ2 / Fishers exact was used for the p-value |
|  | #992 | RR_methd | 4 Ratio of proportions + Chi-squared test | 888 Unclear / Not reported / Unable to determine / Missing | We assume that a crude estimator was used to derive the point estimate, the approach for the 95% CI is unclear, and the χ2 / Fishers exact was used for the p-value |
|  | #1072 | RR_methd | 3 Marginal standardisation | 888 Unclear / Not reported / Unable to determine / Missing | 3 Marginal standardisation |
|  | #474 | RR_unadj_est | 0.78 | 0.79 | 0.78 (ITT) |
|  | #925 | RR_unadj_lci_inf_rprt | 0 No | 1 Yes | 0 No |
|  | #992 | RR_unadj_lci_inf_rprt | 0 No | 1 Yes | 0 No |
|  | #474 | RR_unadj_uci | 1.17 | 1.19 | 1.17 (ITT) |
|  | #992 | RR_unadj_pval_rprt | 1 Yes | 888 Unclear / Not reported / Unable to determine / Missing | 1 Yes |
|  | #232 | cov_adjrr | 0 No | 1 Yes | 0 No |
|  | #232 | tot_no_cov_arr_rprt | 0 No | 1 Yes | 0 No |
|  | #232 | tot_no_cov_arr | 0 | 3 | 3 |
|  | #474 | tot_no_cov_arr | 1 | 2 | 2 |
|  | #1072 | tot_no_cov_arr | 4 | 3 | 3 |
|  | #232 | reason_aRR | 1 Pre­specification (improved efficiency with prognostic factors) | 888 Unclear / Not reported / Unable to determine / Missing | We assume that adjusting for baseline covariates implies pre-specification, which in turn implies improved efficiency. |
|  | #474 | reason_aRR | 1 Pre­specification (improved efficiency with prognostic factors) | 2 Data-driven ­ post-hoc (e.g., lack of balance / statistical significance) | 2 Data-driven ­ post-hoc (e.g., lack of balance / statistical significance) |
|  | #474 | aRR_anlys | 3 Exploratory analysis | 4 Sensitivity analysis | 4 Sensitivity analysis |
|  | #1072 | aRR_anlys | 1 Primary analysis | 4 Sensitivity analysis | 4 Sensitivity analysis |
|  | #232 | aRR_methd | 999 Other (specify) | 2 Modified Poisson | 2 Modified Poisson |
|  | #474 | RR_adj_est | 0.78 | 0.79 | 0.78 (ITT) |
|  | #474 | RR_adj_uci | 1.17 | 1.18 | 1.17 (ITT) |
|  | #232 | RR_adj_pval | 1e-04 | 0.001 | 0.001 |
|  | #918 | RD_rprt | 1 Yes | 0 No | 1 Yes |
|  | #47 | RD_methd | 999 Other (specify) | 888 Unclear / Not reported / Unable to determine / Missing | We assume that a crude estimator was used to derive the point estimate, the approach for the 95% CI is unclear, and the χ2 / Fishers exact was used for the p-value. |
|  | #112 | RD_methd | 4 Difference in proportions +Chi-squared test | 888 Unclear / Not reported / Unable to determine / Missing | We assume that a crude estimator was used to derive the point estimate, the approach for the 95% CI is unclear, and the χ2 / Fishers exact was used for the p-value. |
|  | #210 | RD_methd | 999 Other (specify) | 888 Unclear / Not reported / Unable to determine / Missing | We assume that a crude estimator was used to derive the point estimate, the approach for the 95% CI is unclear, and the χ2 / Fishers exact was used for the p-value. |
|  | #232 | RD_methd | 999 Other (specify) | 888 Unclear / Not reported / Unable to determine / Missing | We assume that a crude estimator was used to derive the point estimate, the approach for the 95% CI is unclear, and the χ2 / Fishers exact was used for the p-value. |
|  | #295 | RD_methd | 4 Difference in proportions +Chi-squared test | 888 Unclear / Not reported / Unable to determine / Missing | We assume that a crude estimator was used to derive the point estimate, the approach for the 95% CI is unclear, and the χ2 / Fishers exact was used for the p-value. |
|  | #360 | RD_methd | 999 Other (specify) | 888 Unclear / Not reported / Unable to determine / Missing | We assume that a crude estimator was used to derive the point estimate, the approach for the 95% CI is unclear, and the χ2 / Fishers exact was used for the p-value. |
|  | #474 | RD_methd | 999 Other (specify) | 888 Unclear / Not reported / Unable to determine / Missing | We assume that a crude estimator was used to derive the point estimate, the approach for the 95% CI is unclear, and the χ2 / Fishers exact was used for the p-value. |
|  | #907 | RD_methd | 999 Other (specify) | 888 Unclear / Not reported / Unable to determine / Missing | We assume that a crude estimator was used to derive the point estimate, the approach for the 95% CI is unclear, and the χ2 / Fishers exact was used for the p-value. |
|  | #992 | RD_methd | 4 Difference in proportions +Chi-squared test | 888 Unclear / Not reported / Unable to determine / Missing | We assume that a crude estimator was used to derive the point estimate, the approach for the 95% CI is unclear, and the χ2 / Fishers exact was used for the p-value. |
|  | #1068 | RD_methd | 4 Difference in proportions +Chi-squared test | 888 Unclear / Not reported / Unable to determine / Missing | We assume that a crude estimator was used to derive the point estimate, the approach for the 95% CI is unclear, and the χ2 / Fishers exact was used for the p-value. |
|  | #1072 | RD_methd | 3 Marginal standardisation | 888 Unclear / Not reported / Unable to determine / Missing | 3 Marginal standardisation |
|  | #295 | RD_unadj_est | -2.6 | 14 | 14 |
|  | #474 | RD_unadj_est | -3.1 | -3 | -3.1 (ITT) |
|  | #295 | RD_unadj_lci | -7.3 | 8 | 8 |
|  | #112 | RD_unadj_uci | 0.2 | -0.2 | -0.2 |
|  | #295 | RD_unadj_uci | 2.2 | 20 | 20 |
|  | #47 | RD_unadj_pval_rprt | 888 Unclear / Not reported / Unable to determine / Missing | 0 No | 0 No |
|  | #210 | RD_unadj_pval_rprt | 1 Yes | 0 No | 888 Unclear / Not reported / Unable to determine / Missing |
|  | #232 | RD_unadj_pval_rprt | 888 Unclear / Not reported / Unable to determine / Missing | 0 No | 0 No |
|  | #360 | RD_unadj_pval_rprt | 0 No | 888 Unclear / Not reported / Unable to determine / Missing | 0 No |
|  | #670 | RD_unadj_pval_rprt | 888 Unclear / Not reported / Unable to determine / Missing | 0 No | 888 Unclear / Not reported / Unable to determine / Missing |
|  | #980 | RD_unadj_pval_rprt | 1 Yes | 0 No | 888 Unclear / Not reported / Unable to determine / Missing |
|  | #992 | RD_unadj_pval_rprt | 1 Yes | 888 Unclear / Not reported / Unable to determine / Missing | 888 Unclear / Not reported / Unable to determine / Missing |
|  | #295 | RD_unadj_pval | 0.27 | 0.001 | 0.001 |
|  | #47 | cov_adjrd | 0 No | 1 Yes | 1 Yes |
|  | #521 | cov_adjrd | 0 No | 1 Yes | 1 Yes |
|  | #943 | cov_adjrd | 1 Yes | 888 Unclear / Not reported / Unable to determine | 888 Unclear / Not reported / Unable to determine |
|  | #47 | tot_no_cov_ard_rprt | 0 No | 1 Yes | 1 Yes |
|  | #521 | tot_no_cov_ard_rprt | 0 No | 1 Yes | 1 Yes |
|  | #47 | tot_no_cov_ard | 0 | 2 | 2 |
|  | #521 | tot_no_cov_ard | 0 | 5 | 5 |
|  | #907 | tot_no_cov_ard | 2 | 3 | 3 |
|  | #918 | tot_no_cov_ard | 2 | 5 | 2 |
|  | #1072 | tot_no_cov_ard | 4 | 3 | 3 |
|  | #47 | reason_aRD | 1 Pre­specification (improved efficiency with prognostic factors) | 888 Unclear / Not reported / Unable to determine / Missing | We assume that adjusting for baseline covariates implies pre-specification, which in turn implies improved efficiency |
|  | #521 | reason_aRD | 1 Pre­specification (improved efficiency with prognostic factors) | 999 Other (specify) | 2 Data-driven ­ post-hoc (e.g., lack of balance / statistical significance) |
|  | #907 | reason_aRD | 1 Pre­specification (improved efficiency with prognostic factors) | 888 Unclear / Not reported / Unable to determine / Missing | We assume that adjusting for baseline covariates implies pre-specification, which in turn implies improved efficiency |
|  | #918 | reason_aRD | 1 Pre­specification (improved efficiency with prognostic factors) | 888 Unclear / Not reported / Unable to determine / Missing | We assume that adjusting for baseline covariates implies pre-specification, which in turn implies improved efficiency |
|  | #943 | reason_aRD | 1 Pre­specification (improved efficiency with prognostic factors) | 888 Unclear / Not reported / Unable to determine / Missing | We assume that adjusting for baseline covariates implies pre-specification, which in turn implies improved efficiency |
|  | #1049 | reason_aRD | 1 Pre­specification (improved efficiency with prognostic factors) | 0 No | We assume that adjusting for baseline covariates implies pre-specification, which in turn implies improved efficiency. |
|  | #1106 | reason_aRD | 888 Unclear / Not reported / Unable to determine / Missing | 0 No | 888 Unclear / Not reported / Unable to determine / Missing |
|  | #47 | aRD_anlys | 1 Primary analysis | 888 Unclear / Not reported / Unable to determine / Missing | 1 Primary analysis |
|  | #907 | aRD_anlys | 1 Primary analysis | 4 Sensitivity analysis | 4 Sensitivity analysis |
|  | #1072 | aRD_anlys | 1 Primary analysis | 4 Sensitivity analysis | 4 Sensitivity analysis |
|  | #943 | aRD_methd | 999 Other (specify) | 888 Unclear / Not reported / Unable to determine / Missing | 888 Unclear / Not reported / Unable to determine / Missing |
|  | #1106 | aRD_methd | 999 Other (specify) | 1 Binomial model, identity link | 1 Binomial model, identity link |
|  | #907 | RD_adj_est | 21.7 | 22.3 | 21.7 (ITT) |
|  | #907 | RD_adj_lci | 12.4 | 12.9 | 12.4 (ITT) |
|  | #907 | RD_adj_uci | 29.8 | 30.4 | 29.8 (ITT) |
|  | #47 | RD_adj_pval_rprt | 888 Unclear / Not reported / Unable to determine / Missing | 0 No | 0 No |
|  | #907 | RD_adj_pval_rprt | 0 No | 1 Yes | 1 Yes |

***Footnotes****: No. – Number; JT - Jacqueline Thompson; LM – Lee Middleton; χ2 - Chi-squared; CI - Confidence Interval; ITT - Intention-To-Treat*

*Table A5: Detailed description of methods for estimating relative risks from overall and nested sample*

| **Analysis approach** | **Unadjusted RR**  **N= 96** | | **Analysis approach** | **Adjusted RR**  **N= 82** | |
| --- | --- | --- | --- | --- | --- |
|  | **N (%)** | **95% CI** |  | **N (%)** | **95% CI** |
| **Other methods** |  |  | **Other methods** |  |  |
| Miettinen-Nurmimen, Fishers exact test | 1 (1%) | <1%, 6% | Inverse probability weighting | 1 (1%) | <1%, 8% |
| Non-parametric bootstrap, permutation test of 10000 iterations | 1 (1%) | <1%, 6% | Inverse variance weighted average | 1 (1%) | <1%, 8% |
| Wald likelihood ratio approximation test, Chi-square test | 2 (2%) | <1%, 8% | Substitution method | 1 (1%) | <1%, 8% |
| Z-test, normal approximation CI | 1 (1%) | <1%, 6% |  |  |  |
|  |  |  |  |  |  |
| **Common methods with some modification** |  |  | **Common methods with some modification** |  |  |
| Log-binomial model | 21 (22%) | 14%, 32% | Log-binomial model | 28 (34%) | 24%, 46% |
| Marginal standardisation | 1 (1%) | <1%, 6% | *Log-binomial model with Firth correction* | 1 (1%) | <1%, 8% |
| Modified Poisson | 8 (8%) | 4%, 16% | GEE | 11 (13%) | 7%, 23% |
|  |  |  | Marginal standardisation | 2 (2%) | <1%, 9% |
|  |  |  | Modified Poisson | 19 (23%) | 15%, 34% |
|  |  |  | *Modified Poisson GEE* | 2 (2%) | <1%, 9% |
|  |  |  |  |  |  |
| **Unclear approaches** |  |  | **Unclear approaches** |  |  |
| Unclear^1^ | 2 (2%) | <1%, 8% | Unclear | 6 (7%) | 3%, 16% |
| Chi-squared test^2^ | 40 (42%) | 32%, 52% | Chi-squared test | 2 (2%) | <1%, 9% |
| Cochran-Mantel-Haenszel test^2^ | 6 (6%) | 3%, 14% | Cochran-Mantel-Haenszel test | 7 (9%) | 4%, 17% |
| Fishers exact test^2^ | 13 (14%) | 8%, 22% | Fishers exact test | 1 (1%) | <1%, 8% |

***Footnotes****: N – Number; CI - Confidence Interval; GEE - Generalised Estimating Equation; GLM - Generalised Linear Model; Z – Z-score.*

^1^Unclear represents situations where no information was provided or the information was unclear, not reported, unable to be determined, or missing.

^2^We assume that a crude estimator is used for the RR, but this was not explicitly reported in the RCTs included in this review.

| *Table A6: Detailed description of methods for estimating risk differences from overall and nested sample* | | | | | |
| --- | --- | --- | --- | --- | --- |
| **Analysis approach** | **Unadjusted RD**  **N = 194** | | **Analysis approach** | **Adjusted RD**  **N = 92** | |
| **Other methods** | **N (%)** | **95% CI** | **Other methods** | **N (%)** | **95% CI** |
| Bayesian bivariate model | 1 (<1%) | <1%, 3% | Bayesian model | 1 (1%) | <1%, 7% |
| *Bayesian model* | 2 (1%) | <1%, 4% | *Bayesian, bivariate model* | 1 (1%) | <1%, 7% |
| Blackwelders equivalence testing, Miettinen–Nurminen method | 1 (<1%) | <1%, 3% | Beach-Meier approach | 1 (1%) | <1%, 7% |
| Cochran-Mantel-Haenszel, Sato variance estimator Wald CI | 1 (<1%) | <1%, 3% | Inverse probability weighting | 1 (1%) | <1%, 7% |
| Continuity corrected Wilson score method | 1 (<1%) | <1%, 3% | Standardised estimator, bootstrapped CI | 1 (1%) | <1%, 7% |
| Crude estimator, Newcombe method | 1 (<1%) | <1%, 3% | Targeted Maximum Likelihood Estimator^4^ | 1 (1%) | <1%, 7% |
| Exact Binomial CI | 1 (<1%) | <1%, 3% | Wilson score method | 1 (1%) | <1%, 7% |
| *Exact Binomial CI, Exact Z test* | 2 (1%) | <1%, 4% |  |  |  |
| Farrington–Manning method, 1-sided 95% CI to assess non-inferiority | 5 (3%) | <1%, 6% |  |  |  |
| Frequentist and Bayesian framework | 1 (<1%) | <1%, 3% |  |  |  |
| Miettinen-Nurminen method | 4 (2%) | <1%, 6% |  |  |  |
| *Miettinen-Nurmimen method, Fishers exact test* | 1 (<1%) | <1%, 3% |  |  |  |
| Newcombe method, continuity correction | 1 (<1%) | <1%, 3% |  |  |  |
| *Newcombe method, Wald test* | 1 (<1%) | <1%, 3% |  |  |  |
| Shan and Wang method, Exact CI | 1 (<1%) | <1%, 3% |  |  |  |
| Targeted Maximum Likelihood Estimator, Chi-square test | 1 (<1%) | <1%, 3% |  |  |  |
| Wald CI | 4 (2%) | <1%, 6% |  |  |  |
| *Wald method, continuity correction* | 1 (<1%) | <1%, 3% |  |  |  |
| Wilson score method | 2 (1%) | <1%, 4% |  |  |  |
| *Wilson score method, Fisher’s exact test* | 1 (<1%) | <1%, 3% |  |  |  |
| *Wilson score method, Z test, Chi-square/Fishers exact test* | 1 (<1%) | <1%, 3% |  |  |  |
|  |  |  |  |  |  |
| **Common methods with some modification** |  |  | **Common methods with some modification** |  |  |
| Binomial model, identity link | 6 (3.1%) | <1%, 7% | Binomial model, identity link | 18 (20%) | 14%, 32% |
| *Binomial model, identity link, GEE* | 3 (2%) | <1%, 5% | *Binomial model, identity link, GEE* | 9 (10%) | 5%, 18% |
| Linear model | 4 (2%) | <1%, 6% | Linear model | 6 (6%) | 3%, 14% |
| *Linear model, Permutation test based on 10,000 replicates^1^* | 1 (<1%) | <1%, 3% | Marginal standardisation | 12 (13%) | 7%, 22% |
| *Least Squares Means, Clopper-Pearson exact CI* | 1 (<1%) | <1%, 3% | Modified Poisson, identity link | 2 (2%) | <1%, 8% |
| Marginal standardisation | 4 (2%) | <1%, 6% | *Modified Poisson, identity link, GEE* | 2 (2%) | <1%, 8% |
| Modified Poisson, identity link | 1 (<1%) | <1%, 3% |  |  |  |
| *Modified Poisson, identity link, GEE* | 1 (<1%) | <1%, 3% |  |  |  |
|  |  |  |  |  |  |
| **Unclear approaches** |  |  | **Unclear approaches** |  |  |
| Unclear^1^ | 33 (17%) | 12%, 23% | Unclear | 12 (13%) | 7%, 22% |
| Chi-squared test | 43 (22%) | 17%, 29% | Chi-squared/Fisher’s exact test | 2 (2%) | <1%, 8% |
| *Chi-squared test, Yates continuity correction* | 2 (1%) | <1%, 4% | Cochran-Mantel-Haenszel test | 20 (22%) | 14%, 32% |
| *Chi-squared/Fisher’s exact test* | 25 (13%) | 9%, 19% | *Cochran-Mantel-Haenszel test, Fisher’s exact test* | 1 (1%) | <1%, 7% |
| Cochran-Mantel-Haenszel test | 14 (7%) | 4%, 12% | *Cochran-Mantel-Haenszel, Chi-squared test* | 1 (1%) | <1%, 7% |
| Crude estimator^2^ | 6 (3%) | <1%, 7% |  |  |  |
| Fishers exact test | 12 (6%) | 3%, 11% |  |  |  |
| Wald LHR approximation test, Chi-square test | 1 (<1%) | <1%, 3% |  |  |  |
| *Wald test* | 1 (<1%) | <1%, 3% |  |  |  |
| *Z test, Chi-square/Fishers exact test* | 1 (<1%) | <1%, 3% |  |  |  |
| *Z test, normal approximation* | 1 (<1%) | <1%, 3% |  |  |  |

***Footnotes:*** *RD – Risk Difference; No – Number; CI - Confidence Interval; GEE – Generalised Estimating Equation; tMLE - targeted maximum likelihood estimator; NA – Not applicable. ^1^Unclear represents situations where no information was provided, the information was unclear, unable to be determined, or missing. ^2^2-by-2 table*
